# Dysregulation of Astrocytic ATP/Adenosine Release in the Hippocampus Cause Cognitive and Affective Disorders: Molecular Mechanisms, Diagnosis, and Therapy

**DOI:** 10.1002/mco2.70177

**Published:** 2025-04-17

**Authors:** Peter Illes, Patrizia Rubini, Henning Ulrich, Hai‐Yan Yin, Yong Tang

**Affiliations:** ^1^ International Research Center on Purinergic Signaling School of Acupuncture and Tuina Chengdu University of Traditional Chinese Medicine Chengdu China; ^2^ Rudolf Boehm Institute for Pharmacology and Toxicology University of Leipzig Germany; ^3^ Acupuncture and Chronobiology Key Laboratory of Sichuan Province Chengdu China; ^4^ Department of Biochemistry Institute of Chemistry University of São Paulo São Paulo Brazil; ^5^ School of Health and Rehabilitation Chengdu University of Traditional Chinese Medicine Chengdu China

**Keywords:** A2A receptors, adenosine, astrocytic ATP, cognitive disorders, depressive‐like behavior, P2X receptors, P2Y receptors

## Abstract

The gliotransmitter adenosine 5'‐triphosphate (ATP) and its enzymatic degradation product adenosine play a major role in orchestrating in the hippocampus cognitive and affective functions via P2 purinoceptors (P2X, P2Y) and P1 adenosine receptors (A1, A2A). Although numerous reviews exist on purinoceptors that modulate these functions, there is an apparent gap relating to the involvement of astrocyte‐derived extracellular ATP. Our review focuses on the following issues: An impeded release of ATP from hippocampal astrocytes through vesicular mechanisms or connexin hemichannels and pannexin channels interferes with spatial working memory in rodents. The pharmacological blockade of P2Y1 receptors (P2Y1Rs) reverses the deficits in learning/memory performance in mouse models of familial Alzheimer's disease (AD). Similarly, in mouse models of major depressive disorder (MDD), based on acute or chronic stress‐induced development of depressive‐like behavior, a reduced exocytotic/channel‐mediated ATP release from hippocampal astrocytes results in the deterioration of these behavioral responses. However, on the opposite, the increased stimulation of the microglial/astrocytic P2X7R‐channel by ATP causes neuroinflammation and in consequence depressive‐like behavior. In conclusion, there is strong evidence for the assumption that gliotransmitter ATP is intimately involved in the pathophysiology of cognitive and affective neuron/astrocyte‐based human illnesses opening new diagnostic and therapeutic vistas for AD and MDD.

## Graphical abstract

Reactive astrocytes release various molecules, which play a role in maintaining central nervous system (CNS) homeostasis. (1) Gliotransmitters, such as glutamate, γ‐aminobutyric acid (GABA), ATP, and D‐serine, are secreted by vesicular exocytosis. In addition, astrocytes can also release ATP and glutamate through (2) P2X7Rs, (3) connexin‐43 (Cx43) hemichannels and pannexin‐1 (Panx‐1) channels. (4) Prostanoid,. reactive oxygen (ROS) and nitrogen (RNS) species also leave the intracellular space. (5) Proteases such as caspase‐1 are outpoured to degrade various proteins. (6) The proinflammatory cytokines interleukin‐1β (IL‐1β), IL‐6, and IL‐18, as well as tumor necrosis factor‐α (TNF‐α) cause neuroinflammation. Damage to CNS cells contributes to neurodegeneration and promotes cognitive impairment, for example, in Alzheimer's disease (AD). (7) However, reactive astrocytes also secrete the growth factors brain‐derived neurotrophic factor (BDNF), glial‐cell‐derived neurotrophic factor (GDNF), and nerve growth factor (NGF), promoting proliferation. Reactive astrocytes promote neuroinflammation, which is a cause of major depression in humans and depressive‐like behavior in rodents. Reproduced with permission from Ref. [259].

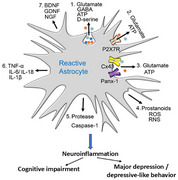

## 1 Introduction

The major non‐neuronal cell population of the CNS consists of glial cells such as astrocytes, oligodendrocytes, and microglia. These cell types interact with neurons to shape various functions of the brain and spinal cord. In short, astrocytes appear to be important players in serving ionic and neurotransmitter homeostasis with special emphasis on the clearance of extracellular potassium and glutamate [1, 2]. In addition, after the release of glutamate/GABA from neurons and subsequent uptake into astrocytes, these cells synthesize and release glutamine, which is taken up into neurons and is used as a neurotransmitter precursor for the production of glutamate and GABA [3]. Astrocytes enwrapping synapses also control the extracellular space volume and hence the extracellular concentrations and diffusion of neuroactive substances [4]. Besides these homeostatic functions, astrocytes are endowed with a range of neurotransmitter receptors, whose activation may result in elaborate [Ca^2+^]_i_ transients to induce the release of gliotransmitters (ATP, glutamate, D‐serine) stimulating their receptors at neuronal terminals/cell bodies and establishing thereby a modulation of synaptic functions [5, 6]. The functional entity of pre‐ and postsynaptic neurons as well as the involved astrocytic processes are termed the “tripartite synapse” giving credit to the ability of astrocytes to modulate neuronal functions [7].

Eventually, astrocytes mediate neurovascular signaling to capillary pericytes in order to cause vasodilation via the arachidonic acid metabolites prostaglandin (PG) E_2_ and 20‐hyroxyeicosatetraenoic acid [8]. In sleep‐promoting neurons of the ventrolateral preoptic nucleus, PGD_2_Rs are expressed in astrocytes mediating increased adenosine release, which exerts on its behalf a control of local blood flow [9]. Adenosine appeared to be involved also in the blood oxygenation level‐dependent functional magnetic resonance imaging signals of the rat somatosensory cortex during periods of enhanced neuronal activity [10]. It has been suggested that during exacerbated neuronal firing, astrocytes respond to neuronal ATP, which propagates astrocytic activation, stimulates release of vasoactive substances (e.g., adenosine), and thereby dilation of cerebral vasculature.

ATP is intimately involved in the pathophysiology of cognitive and affective neuron/astrocyte‐based illnesses. In this respect, the subject of our overview is defined by the title, which points out a restriction to the emotional and cognitive functions of ATP released by astrocytes and also to similar functions of adenosine generated by the enzymatic degradation of astrocytic ATP. Release means in our understanding an active process, such as Ca^2+^‐dependent exocytosis, passage through (hemi)channels after their opening, and operation of equilibrative nucleoside transporters (ENT), but by no‐way passive outflow from damaged cells (see Section 2.1). Thus, we concentrate ourselves on astrocytic, but not neuronal release of ATP and adenosine, the latter of which is generated from this ATP via enzymatic decomposition or is alternatively driven out of cells by a concentration‐dependent transporter. This limitation is necessary because otherwise the review would include a considerable amount of data about ATP/adenosine and their receptors published over many years, without giving consideration to these specific issues raised.

As indicated above, our aim was to summarize the reported effects of astrocyte‐derived ATP (and its enzymatic degradation product adenosine) on cognitive and affective functions and their disturbances in the respective rodent models. It is expected that these considerations will have diagnostic and therapeutic consequences especially for Alzheimer's disease (AD) and major depressive disorder (MDD) in human medicine.

## 2 Astrocytic ATP and its Enzymatic Degradation Product Adenosine

### 2.1 Astrocyte‐Derived Release of ATP and its Degradation to Adenosine

A manipulation that interferes with astrocyte vesicular release of gliotransmitters via overexpression of a dominant‐negative domain of vesicular *s*oluble *N*‐ethylmaleimide‐sensitive‐factor attachment *re*ceptor (dnSNARE) has led to the realization of astrocytic involvement in processes that were traditionally considered strictly neuronal, including the sleep–wake cycle, long‐term potentiation (LTP), and cognition [11]. Although the astrocyte specificity of this manipulation was questioned later, by demonstrating widespread expression of the *dnSNARE* transgene in cortical neurons, and the finding that the activity of cortical neurons is reversibly suppressed in dnSNARE mice [12], these animals are still widely used in order to demonstrate the release of gliotransmitters, such as ATP/adenosine from astrocytes (e.g., [13, 14].

Hence, ATP is besides its general signaling function in the CNS (e.g., outflow from endothelial cells on shear stress from brain capillaries; [15]), also a neurotransmitter, released from synaptic‐type vesicles of nerve terminals [16], and a gliotransmitter, released via exocytotic, Ca^2+^‐ and SNARE complex‐dependent mechanisms from astrocytic lysosomes [17, 18]. While in neurons, the immediate stimulus for exocytotic (vesicular) transmitter release is extracellular Ca^2+^ passing via voltage‐sensitive Ca^2+^ channels the plasma membrane and thereby increasing [Ca^2+^]_i_ in astrocytes, this increase is fueled by the activation of G proteins coupled to the inositol 1,4,5‐triphosphate receptor subtype 2 (IP3R2) release channels, located at the endoplasmic reticulum and triggering [Ca^2+^]_i_ increase [19]. Combined epifluorescence and total internal reflection fluorescence microscopy was used to monitor individual quinacrine‐loaded, ATP‐containing vesicles undergoing exocytosis in cultured astrocytes [20]. Two populations of ATP‐containing vesicles with distinct (fast and slow) time‐course of cargo release were identified. These ATP pools were thought to represent synaptic‐type vesicles and secretory lysosomes in astrocytes [21]; the speed of transmitter release was proven to be at least two orders of magnitude slower than the kinetics of regulated exocytosis recorded in neurons [22]. A prerequisite for ATP release is the active accumulation of this nucleotide into the secretory vesicles/lysosomes by a vesicular nucleotide transporter (VNUT; [23]).

ATP may leave astrocytes also by nonexocytotic pathways, through connexin hemichannels (mostly connexin‐43 [Cx43]; [24]) and pannexin 1 (Panx‐1) channels [25]. Maxi‐anion channels [26], volume‐regulated anion channels [27], the Ca^2+^‐dependent Cl^−^ channel bestrophin [28], the calcium homeostasis modulator 1‐3 (CALHM1‐3; [29]), and the purinergic receptor P2X7R [30, 31] also participate in the accumulation of ATP outside of cells. Hence, neurotransmitters (e.g., noradrenaline) may stimulate their astrocytic receptors, resulting in the subsequent release of the gliotransmitter ATP, which in turn modulates synaptic functions [32, 33].

Extracellular adenosine in the CNS derives from the enzymatic degradation of neuronal and astrocytic ATP by ectonucleotide tri(di)phosphohydrolase (ENTPD; CD39) and the subsequent degradation of AMP by ecto‐5’‐nucleotidase (CD73) [34, 35]. Besides this “conventional” and Ca^2+^‐dependent, although indirect generation of adenosine, the nucleoside may be also outpoured from astrocytes by the operation of ENT1 and 2. There is some evidence for the exocytotic, Ca^2+^‐dependent release of adenosine itself from separate neuronal/astrocytic synaptic vesicles [36, 37], or the Ca^2+^‐dependent operation of ENT‐1 [37], generating increased adenosine concentrations in the extracellular space. These latter release mechanisms are, however, far from being generally accepted as sources of extracellular adenosine.

### 2.2 Astrocytic Purinoceptors

Extracellular ATP released by vesicular and nonvesicular means stimulates ionotropic P2XRs (assembled as trimeric homo‐ or heteromeric receptors from seven subunits, P2X1‐7) and metabotropic P2YRs (eight subtypes, P2Y1, 2, 4, 6, 11, 12, 13, 14; [38, 39]), most of which are localized also at astrocytes [40, 41]. P2XRs open nonselective cationic channels, while P2YRs are coupled to G_q_, G_s_, or G_i/o_ proteins and their obligatory second messenger systems. Whereas P2XRs respond only to ATP [42, 43], P2YRs respond to ATP/ADP, UTP/UDP, or UDP sugars [44, 45]. Adenosine, the enzymatic degradation product of ATP, acts on G protein‐coupled P2Rs (G_s_, G_i/o_), which either stimulate (A2A, A2B) or inhibit (A1, A3) adenylate cyclase [46, 47]. The two behaviorally relevant high‐affinity receptors, A1 and A2A, however, exert their effects not exclusively via the inhibition and stimulation of adenylate cyclase production (see Section 4.7).

Behavioral effects of adenosine are mediated in the CNS both by A1 and A2ARs [48, 49]. Signaling through these receptors is terminated by rapid uptake through the function of ENT1‐2 [50] and to a lesser extent by the conversion of adenosine to the only slightly active inosine in the extracellular space by the already mentioned 5’‐nucleotidase [34, 35] (see in Section 4.6.1). Another possibility of enzymatic conversion of adenosine is that to AMP by adenosine kinase, which is however an intracellular enzyme, unable to bind extracellular adenosine to terminate its action [51]. Astrogliosis, via overexpression of adenosine kinase, induces a deficiency in the homeostatic tone of adenosine, which is a common hallmark of epilepsy, AD, Parkinson's disease (PD), and amyotrophic lateral sclerosis [52].

**FIGURE 1:**
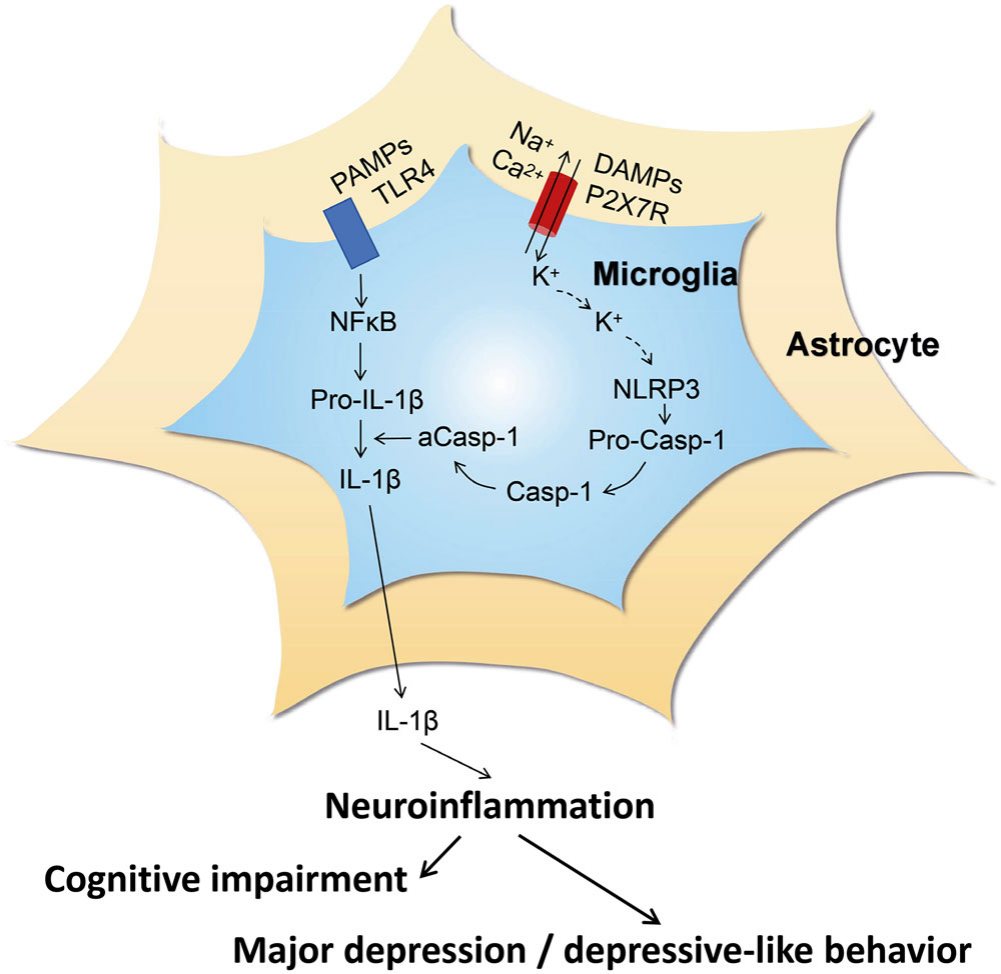
Microglia and astrocytes are sources of proinflammatory cytokines leading to neuroinflammation. The inner cell border in this figure exemplifies a microglial cell, while the outer cell boarder exemplifies an astrocyte. Secretion of interleukin‐1β (IL‐1β) is caused both in microglia and astrocytes by the function of the nucleotide‐binding, leucine‐rich repeat, pyrin domain containing inflammasome‐3 (NLRP3). Pathogen‐associated molecular patterns (PAMPs; e.g., bacterial lipopolysaccharide; LPS) act on toll‐like receptor‐4 (TLR4) and induce its phosphorylation. In consequence, in the cell nucleus, nuclear factor κB (NFκB) is activated, which promotes the synthesis of the NLRP3 inflammasome and pro‐IL‐1β, both accumulating in their inactive forms in the cytosol. The activation of NLRP3 is primarily due to a decrease of the intracellular K^+^ concentration mostly initiated by the efflux of K^+^ via the P2X7R. The next step is degradation of pro‐caspase‐1 to caspase‐1 and then the generation of active caspase‐1 (aCasp‐1), which finally degrades pro‐IL‐β to mature IL‐1β, leaving the cell by a number of mechanisms. This proinflammatory cytokine together with IL‐18 and tumor necrosis factor‐α (TNF‐α), the synthesis of which is also promoted by P2X7Rs, causes neuroinflammation and in consequence cognitive impairment in humans and animals, as well as major depressive disorder (MDD) in humans and depressive‐like behavior in animals, usually in combination with a genetic predisposition in the case of MDD.

The ionotropic P2X7R is activated by concentrations of ATP in the pathologically high micromolar/millimolar range, in contrast to the other P2XR subtypes, which are activated already by lower micromolar ATP concentrations [53, 54]. Further, although P2X7Rs cause nonselective cationic (Na^+^, K^+^, Ca^2+^) currents, when in initial contact with ATP, longer‐lasting or repetitive stimulation by ATP allows the passage of larger molecules (up to 900 Da) through the cell membrane [55]. These pores are believed to play a direct role in apoptosis/pyroptosis and inflammation [56, 57]. The P2X7R is widely expressed by cells of the innate and adaptive immune systems all over the animal kingdom including the human species [56, 57]. P2X7Rs are preferentially located on microglia, the resident macrophages of the brain, but they also occur at astrocytes [56] (Figure 1). P2X7Rs, in association with toll‐like receptors (TLRs), may cause (neuro)inflammation mainly by releasing the cytokines interleukin‐1β (IL‐1β) and tumor necrosis factor‐α (TNF‐α) mediated by the nucleotide‐binding, leucine‐rich repeat, pyrin domain containing (NLRP3) inflammasome [56, 58].

## 3 Regulation of Cognitive and Affective Functions by the Hippocampus

**FIGURE 2:**
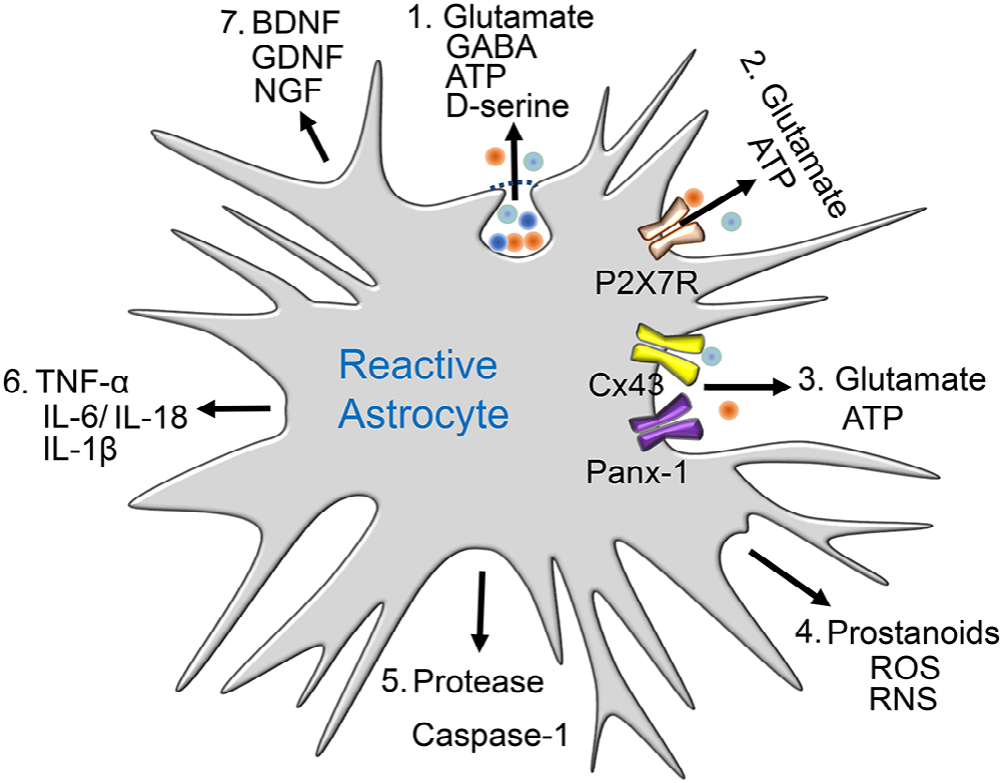
Reactive astrocytes release various molecules that play a role in maintaining CNS homeostasis. (1) Gliotransmitters, such as glutamate, γ‐aminobutyric acid (GABA), ATP, and D‐serine, are secreted by vesicular exocytosis. In addition, astrocytes can also release ATP and glutamate through (2) P2X7Rs, (3) connexin‐43 (Cx43) hemichannels and pannexin‐1 (Panx‐1) channels. (4) Prostanoids, reactive oxygen (ROS) and nitrogen (RNS) species also leave the intracellular space. (5) Proteases such as caspase‐1 are outpoured to degrade various proteins. (6) The proinflammatory cytokines interleukin‐1β (IL‐1β), IL‐6, and IL‐18, as well as tumor necrosis factor‐α (TNF‐α) cause neuroinflammation. Damage to CNS cells contributes to neurodegeneration and promotes cognitive impairment, for example, in Alzheimer's disease (AD). (7) However, reactive astrocytes also secrete the growth factors brain derived neurotrophic factor (BDNF), glial‐cell‐derived neurotrophic factor (GDNF), and nerve growth factor (NGF), promoting proliferation. Reactive astrocytes promote neuroinflammation, which is a cause of major depression in humans and depressive‐like behavior in rodents. Reproduced with permission from Ref. [67].

The hippocampus, as part of the limbic system, is involved in memory, learning (especially spatial learning [59, 60]), and emotion [61, 62]. On the one hand, the hippocampus transfers short‐term memory to long‐term storage; its functions are typically curtailed in illnesses, such as AD or posttraumatic stress disorder. There is broad consensus on the idea that hippocampal LTP and long‐term depression (LTD) are forms of synaptic plasticity, which comprise at the cellular level the physiological correlates of associative learning [63]. On the other hand, emotional behavior is also regulated by the hippocampus, and therefore mood disorders, such as MDD, are thought to be due to disturbed network functions, governed by the hippocampus in association with numerous regions of the brain, for example, the medial or dorsolateral prefrontal cortex (PFC) [64, 65]. In this respect, it should be noted that the hippocampal–prefrontal pathway (a monosynaptic unilateral projection) is highly sensitive to stress, which is a major precipitating factor for the symptoms of depression [66]. The wide range of bioactive molecules released by reactive astrocytes disturbs CNS homeostasis and thereby causes cognitive impairment and major depression (Figure 2).

## 4 Purinergic Regulation of Cognition

### 4.1 Cognitive Disturbances and their Animal Models

Learning is a neural process that enables both humans and animals to adapt to their environment, based on previous experiences; memory is the neural process by which experience acquired during learning is stored and eventually accessed [68]. Memory systems can be classified to declarative and nondeclarative memory [69]. The former can be expressed in words, while the latter encompasses information related to motor skills. Another distinction of cognitive performances is made between long‐term, short‐term, and working memory, based on the duration of time after which previous experiences have to be recalled [70].

Cognitive disorders frequently accompany neurodegenerative disorders, such as AD, PD, stroke, and so on. They may be connected with altered ATP release from astrocytes or altered effects of ATP/ADP at astrocytic purinoceptors [71]. Astrocytes may modify neuronal activities in three different manners. First, astrocytes affect neurons according to their genetic identity—for example, dopamine D1 and D2R‐containing medium spiny neurons (MSNs) are governed differentially [72]. Second, astrocytes exert neurotransmitter‐specific effects on different neuronal types [73]; and third, they exhibit task‐specific effects, for example, depending on the presence or absence of learning processes [74]. Hence, the gliotransmitter ATP and its astrocytic purinoceptors may interfere with the learning ability of rodents and possibly also humans through at least the three above‐cited mechanisms.

Learning/memory disorders may be investigated in various rodent models. The simple and most often used ones are the following: Novel object recognition (recognition memory), T‐maze or Y‐maze (exploratory/spatial memory), Morris water maze (spatial and long‐term reference memory), Barnes maze (spatial learning and memory), passive avoidance (emotional learning and long‐term memory), and contextual fear conditioning (associative context‐driven learning) [75, 76, 77]. Of course, all these test systems only incompletely model the complex cognitive disturbances of humans; nevertheless, they are of considerable help in collecting preclinical evidence and in allowing some extrapolation to patients in the clinical praxis.

### 4.2 Impeded Channel‐Mediated ATP Release from Astrocytes in the Hippocampus is a Cause of Cognitive Impairments

**FIGURE 3:**
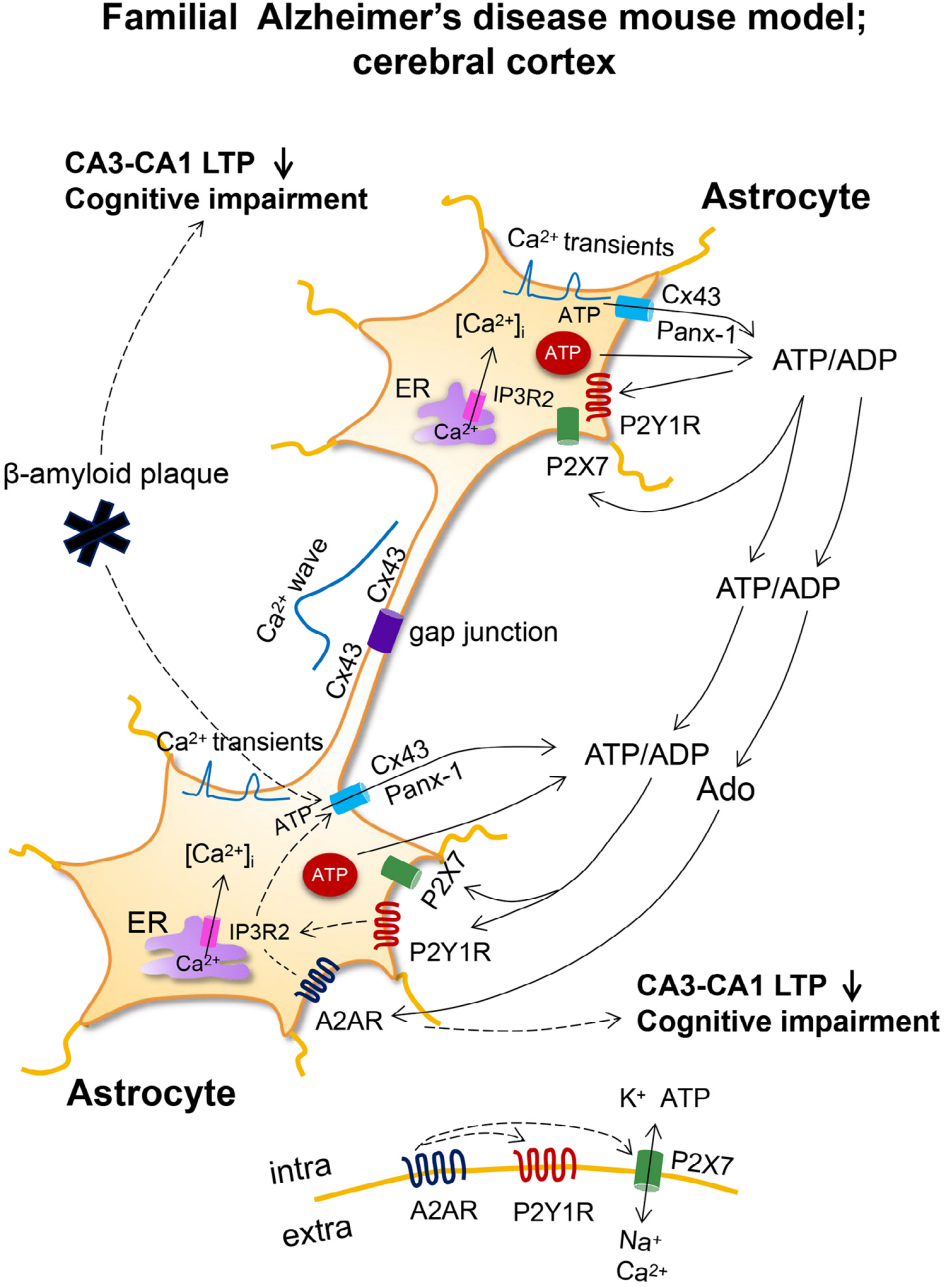
Single cell [Ca^2+^]_i_ transients and oscillations in two hyperactive astrocytes coupled to each other through gap junction channels consisting of two opposed Cx43 hemichannels. In the cerebral cortex of a familial mouse Alzheimer's disease (AD) model, (1) increased astrocytic ATP release induces P2Y1R overstimulation and learning deficit around extracellular β‐amyloid plaques. (2) P2Y1R activation induces the release of Ca^2+^ from the endoplasmic reticulum (ER) via inositol 1,4,5‐triphosphate receptor‐2 (IP3R2). Increased [Ca^2+^]_i_ can be measured by two‐photon microscopy in form of [Ca^2+^]_i_ transients; in addition, this Ca^2+^ flows through gap junction channels to neighboring astrocytes and by means of slower [Ca^2+^]_i_ waves synchronizes astrocytic activities. (3) [Ca^2+^]_i_ transients cause exocytotic (vesicular) ATP secretion into the extracellular space and in addition also nonexocytotic release via Cx43 hemichannels and Panx‐1 channels. The overstimulation of P2Y1Rs by ATP/ADP decreases spatial orientation and memory in the Barnes maze test and the Morris water maze test. (4) Moreover, the rate of rise of the CA3–CA1 LTP is also decreased when compared with normal mice. Both effects can be eliminated and thereby spatial orientation and LTP efficiency can be normalized by pharmacological blockade of P2Y1Rs, the inhibition of the enzymatic degradation of ATP to ADP (the primary agonist of P2Y1Rs), or the genetic deletion of the IP3R2. (5) In addition, ATP is enzymatically degraded to adenosine which by activating presynaptic A1Rs at neurons (see Figure 4), decreases basal synaptic transmission, and by stimulating postsynaptic A2ARs decreases CA3–CA1 LTP amplitude and causes cognitive impairment.

Gap junctions of vertebrates comprise of two opposed connexin hemichannels that link the cytosol of adjacent cells, whereas in invertebrates, they are built up of innexins of related structure [78, 79]. Homologous to innexins are the vertebrate pannexins; however, these do not form gap junctions and exist only in form of channels. As already mentioned earlier, both unopposed connexins and pannexins are release pathways for ATP from astrocytes (Figure 3). Gap junction connections, which ensure the undisturbed network activity of astrocytic populations through the cell‐to‐cell diffusion of Ca^2+^, called Ca^2+^ waves, are also of high physiological significance. Ca^2+^ waves are due to the passive diffusion of Ca^2+^ through the gap junctions, but also to the release of ATP in one astrocyte and the stimulation in the next one of metabotropic P2Y1 and P2Y2Rs releasing Ca^2+^ from the endoplasmic reticulum [80].

There are a number of publications that present evidence on the interrelationship between connexin hemichannel‐ and pannexin channel‐mediated release of ATP from astrocytes, and learning/memory performance of rodents [81] (see below). In particular, Cx43 (a connexin with an approximate molecular weight of 43 kDa) is located on astrocytes in contrast to neurons and has been specifically implicated in activity‐induced heterosynaptic metaplasticity [82].

The microinfusion of transactivator of transcription‐linked Gap19 (TAT‐Gap19) peptide (highly selective to Cx43) into the brain ventricle of mice did not affect the spatial working memory in a spontaneous alternation Y maze task, but impaired the spatial short‐term memory in a delayed spontaneous alternation Y maze task [83]. Along this line of thinking, infrasonic noise impaired cognitive functions as measured in the Morris water maze test; this effect was closely related to the activation of Cx43 hemichannels in astroglia [84]. Blockade of spatial learning/memory by infrasonic noise depended on Cx43, as proven by restitution of cognitive functions after Cx43 blockade with the intrahippocampal injection of shRNA or TAT‐Gap19 peptide targeting this connexin, or alternatively fluorocitrate, a general metabolic toxin of astrocytes.

When the learning/memory capabilities of Panx1^−/−^ mice were compared with their Panx1^+/+^ counterparts, prominent differences were observed [85]. In the novel object recognition and cookie finding tests, the KO mice showed limited performance, confirming the participation of Panx‐1 and the release of gliotransmitters in object recognition and spatial memory. In another study, experiments with a mouse genetically deficient in Panx‐1 documented in the eight arm radial maze that long‐term spatial reference memory, but not spatial working memory was deficient, in comparison with the achievement of the respective control mice [86].

The tight coupling of P2X7Rs with Panx‐1 channels has been verified about two decades ago and it was even concluded that these two channel types act as associated proteins [87, 88]. Hence, Panx‐1 has been assumed to be necessary for the processing of caspase‐1 and the generation of mature IL‐1β induced by P2X7R activation.

Although connexin hemichannels and pannexin channels are known to be expressed both in neurons and astrocytes, it is increasingly believed that P2X7Rs fail to be expressed at neurons; the effects observed appear to be indirect, and due to astrocytic/microglial receptor activation via the release of gliotransmitters/signaling molecules [31, 89, 90, 91].

### 4.3 Anxiety Disorders and Fear Memory

Contextual fear conditioning appears to depend on the hippocampus, basolateral amygdala, and ventromedial PFC [92, 93]. An abundance of data support the assumption that P2X7R antagonists [94] or the genetic deletion of this receptor type [95] interfered in mice with contextual fear recall [96]. When liquid chromatography and mass spectrometry were used to identify substrates of the protein degradation process in the amygdala of male rats following fear conditioning [97], proteins involved in the cytoskeleton, ATP synthesis, and cell signaling were significantly altered during contextual fear acquisition and extinction. Thus, it was suggested that these processes are regulated by P2X7Rs, which open astrocytic Cx43 channels.

In a series of experiments, rats were fear conditioned, using parings of neutral tone (conditional stimulus) with an aversive foot shock (unconditional stimulus; [98]). All animals exhibited equal levels of freezing, when tested 1 day later for their tone fear memories. Intraperitoneal (i.p.) application of the general gap junction blocker carbenoxolone or the selective Cx43 blocker mefloquine significantly reduced context fear. Microinfusion into the rat basolateral amygdala of TAT‐Cx43L2, a peptide that selectively inhibits Cx43 hemichannel opening during memory consolidation, induced amnesia for auditory fear conditioning [99]. Learning capacity was recovered after coinfusion of TATCx43L2 with a mixture of possible gliotransmitters including ATP.

### 4.4 Involvement of P2X and P2Y Receptors in Cognitive Deterioration; AD

Dementia is the leading cause of disability in the elderly population and AD is the most prevalent of all dementias, leading to early deficits in episodic, short‐term memory, followed by progressive impairment in declarative and nondeclarative memory [100, 101]. There is abundant experimental evidence that P2X7R activation and the consequent release of cytokines, chemokines, reactive oxygen/nitrogen species, and the passage through the P2X7R‐channels themselves of the cytotoxic glutamate may cause neurodegeneration superimposed on the primary causes of the disease, which are thought to be the deposition of toxic extra‐ and intracellular protein aggregates (β‐amyloid, hyperphosphorylated tau [79, 102, 103, 104]). We do not discuss these results in detail because there is no definite evidence for the exclusively astrocytic localization of the P2X7R [31, 89].

In vivo two‐photon microscopy demonstrated in astrocytes of the somatosensory cortex the increase of spontaneous [Ca^2+^]_i_ transients in a mouse model of familial AD (APPPS1 mice [105]; Figure 3). This was most pronounced in reactive astrocytes around β‐amyloid plaques and consisted of single‐cell [Ca^2+^]_i_ transients and oscillations. Calcium responses were augmented by increasing ADP (an endogenous agonist of the P2Y1R) levels as consequence of blocking its degradation by apyrase and was reduced by blocking P2Y1Rs with MRS2179 or inhibiting the release of ATP/ADP through connexin hemichannels with carbenoxolone.

In a successor paper of this group of authors, the significance of their findings to the cognitive abilities of the APPPS1 mice was further extended [106]. They documented that the high‐frequency stimulation of Schaffer collaterals induced LTP in the hippocampal CA1 neurons. The extent of LTP was higher in brain slices taken from wild‐type than from the AD‐model animals; however, the application of MRS2179 normalized this difference. Thus, the pathological increase of strength in the CA3–CA1 synapses caused by astrocytic hyperactivity could be normalized by P2Y1R inhibition. In accordance with this cellular model of hippocampal learning, chronic P2Y1R inhibition through application of MRS2179 via osmotic minipumps reversed spatial reference learning and memory deficits in APPPS1 mice. The learning/memory performance was tested in the Barnes maze and Morris water maze tests. Moreover, both elementary memory processes and spatial learning performance could be improved in a mouse model of genetic AD by rescuing normal astrocytic [Ca^2+^]_i_ activity not only by the pharmacological blockade of P2Y1Rs but also by the deletion of metabotropic signaling downstream of P2Y1R activation. This latter effect was achieved by generating mice double transgenic for *Appps1^−/+^* (AD model) and *Ip3r2^−/−^* (blockade of Ca^2+^ release from the endoplasmic reticulum).

As discussed above, astrocytic ATP release via Cx43 channels is a necessary prerequisite for cognitive performance. In apparent disagreement with these findings, in transgenic mice with overexpressed β‐amyloid precursor protein (APP) and presenilin1 (PS1), an increase in astroglial connexin immunoreactivity around the β‐amyloid plaques was documented [107]. Other studies proved the specific expression of astroglial Cx43 around the amyloid plaques of this rodent model of AD [108]. These findings perfectly correlate with the earlier detection of upregulated Cx43‐immunoreactivity in cortical areas of postmortem brains of AD patients [109]. A possible explanation for this apparent controversy is that the increased density of Cx43 around the amyloid plaques is not a reason but a compensatory (maladaptive) consequence of the AD‐induced functional limitation.

### 4.5 P2X and P2Y Receptor‐Mediated Changes in Synaptic Plasticity Events

**FIGURE 4:**
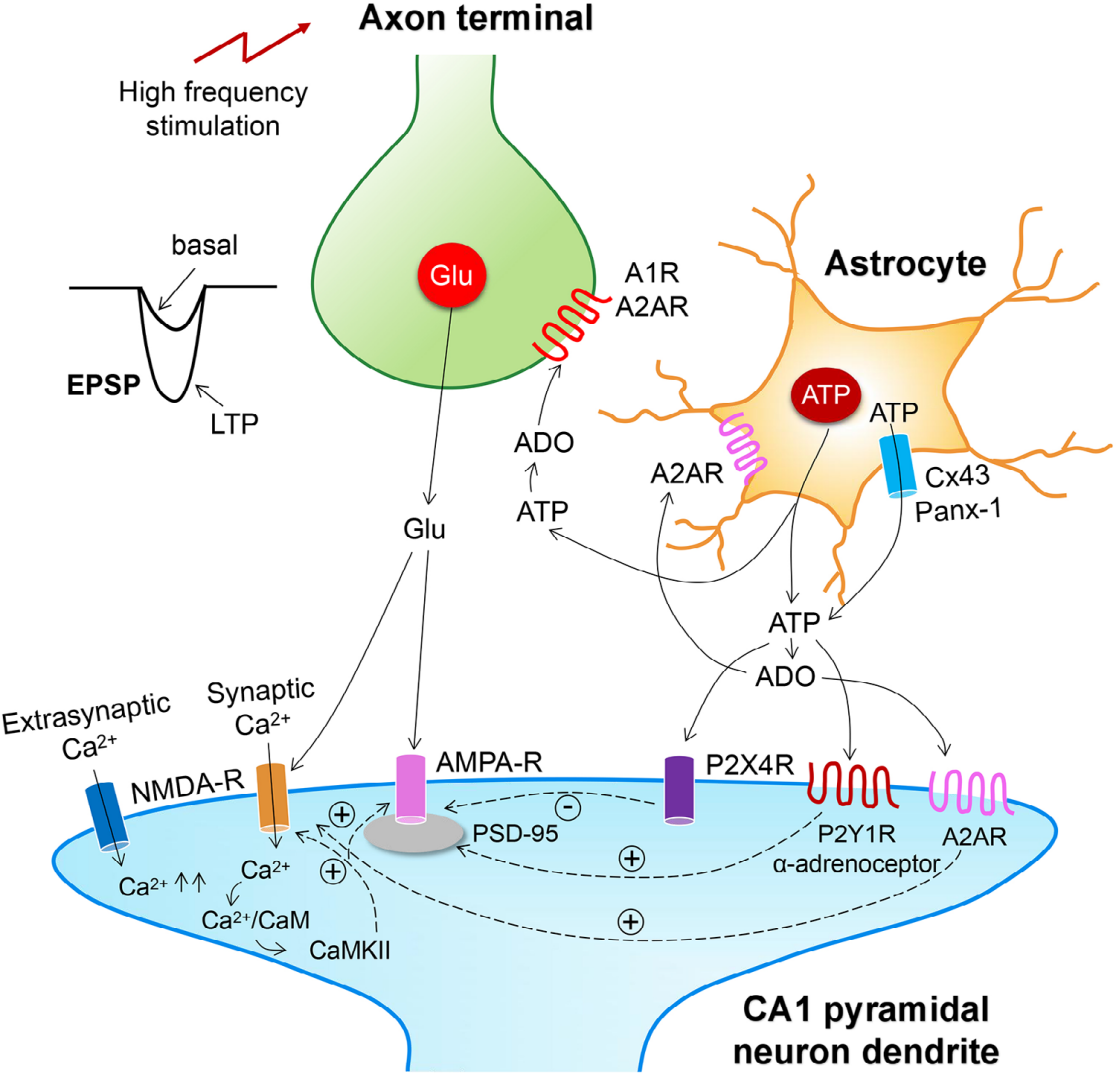
Long‐term potentiation (LTP) in the hippocampal CA3–CA1, or dentate gyrus (DG)–CA3 synapses and their modulation by astrocytic ATP/adenosine. Axonal projections of CA3 pyramidal neurons (Schaffer collaterals) projecting to CA1 pyramidal neurons form synapses with the CA1 dendritic arborization. (1) High frequency electrical stimulation of the Schaffer collaterals causes long‐lasting increase in synaptic strength of the CA3–CA1 synapses, termed long‐term potentiation (LTP). This is due to the release of glutamate onto postsynaptic NMDA‐Rs and the influx of Ca^2+^ through these ionotropic receptors [115]. Then, Ca^2+^ binds to calmodulin (CaM), and Ca^2+^/CaM activates Ca^2+^/calmodulin‐dependent protein kinase II (CaMKII), which binds to the NMDA‐R, maintaining its activity and increasing the incorporation of AMPA‐Rs into the synaptic membrane. (2) ATP is released from astrocytes both by exocytotic/vesicular mechanisms and via Cx43/Panx‐1 hemichannels. This ATP is rapidly degraded by ecto‐nucleotidases and 5′‐nucleotidase to adenosine. (3) Adenosine might activate A1 and A2ARs located at the glutamatergic axon terminals of CA3 pyramidal cells by inhibiting and promoting glutamate release, respectively. (4) On the other hand, ATP might activate postsynaptic P2Y12Rs located at the CA1 pyramidal cell dendrites. The stimulation of postsynaptic α1‐adrenoceptors together with P2Y12R stimulation was shown to favor LTP. The arrow from the two receptors is pointing to the AMPA‐R, indicating the net potentiation of the LTP, although there is probably a direct interaction through the NMDA‐R, because the CA3–CA1–LTP is thought to involve NMDA‐Rs [116]. However, there is no direct experimental evidence for this assumption. (5) The stimulation of postsynaptic A2ARs facilitates synaptic NMDA‐Rs and thereby indirectly supports the LTP [117, 118]. (6) By contrast, the stimulation of extrasynaptic NMDA‐Rs promotes a massive Ca^2+^ influx via these receptors, and in consequence leads to Ca^2+^ overload in the neuron and the activation of apoptotic enzyme cascades. (7) The activation of postsynaptic P2X4Rs might inhibit LTP. The genetic deletion of postsynaptic density protein‐95 (PSD‐95) interferes with the inhibitory interaction between P2X4Rs and the increased incorporation of AMPA‐Rs into the cell membrane. The importance of astrocytic ATP for a full blown LTP is proven by its depression following treatment of brain slices with the astrocytic toxin α‐aminoadipate [119].

P2X and P2YRs have been shown to participate in synaptic plasticity events in the CNS, including cognition‐relevant areas such as the hippocampus, and thereby were found to modulate learning and memory [33, 110, 111, 112] (Figure 4). It has been especially interesting to appreciate that downregulation of NMDAR‐mediated signaling by the P2XR can have significant impact on LTP in the hippocampal CA1 area and the layer 2/3 pyramidal cells of the somatosensory cortex [113, 114].

The combination of electrophysiological recording techniques and multiphoton fluorescent Ca^2+^ imaging in acutely isolated astrocytes from the somatosensory cortex showed that α1‐adrenoceptor activation stimulates the vesicular release of ATP from astrocytes; this initiates a burst of P2XR‐mediated currents in adjacent pyramidal neurons [14]. Such purinergic currents were depressed by intracellular perfusion of astrocytes with the exocytosis inhibitory tetanus toxin. Weak sub‐threshold electrical stimulation can induce LTP, when astrocytes are additionally activated by the preferentially α1‐agonistic noradrenaline. Treatment of the brain slices with tetanus toxin, or preparing the brain slices from dnSNARE mice with decreased ATP release from astrocytes abolished and depressed the induction of LTP, respectively. An alternative to α1‐adrenoceptor‐stimulation to increase intracellular [Ca^2+^]_i_ in astrocytes was the application of ryanodine to induce the release of Ca^2+^ from its storage sites [120]. This procedure also enhanced LTP in the hippocampus and neocortex.

Heterosynaptic (h)LTD at untetanized synapses accompanying the induction of LTP, spatially sharpens the synaptic potentiation. It was found that hLTD in the hippocampal CA1 area is caused by stimulation‐induced ATP release from astrocytes that suppresses transmitter release from untetanized synaptic terminals via activation of P2YRs [121]. P2YR activation was blocked by Reactive Blue‐2, which at that time was thought to selectively inhibit P2YRs (this unfortunately did not prove to be true later) and more reliably by buffering astrocyte [Ca^2+^]_i_ at a low level. The same methodological repertoire described above, allowed the group of Yuriy Pankratov to report that in the neocortex, ATP and glutamate are coreleased onto adjacent pyramidal neurons, and postsynaptic NMDARs are downregulated by simultaneously activated P2XRs [114]. Genetic deletion of postsynaptic density protein‐95 (PSD‐95) abrogated the P2XR‐mediated downregulation of NMDARs. Pharmacological blockade of purinergic modulation of NMDARs by the P2X4R antagonist 5‐BDBD in PSD‐95‐deleted mutant mice, dramatically decreased the threshold of LTP induction and increased the net magnitude of LTP. This is in perfect agreement with the reported finding that recombinant P2X4Rs interact with NMDARs when coexpressed in *Xenopus laevis* oocytes [122].

### 4.6 Changes in Extracellular Adenosine Levels are Causes of Cognitive Deterioration

#### 4.6.1 Changes of Adenosine Levels Arising from the Operation of an ENT are Causes of Cognitive Deterioration

A subgroup of astrocytes in the dorso‐medial striatum regulate the transition from habitual to goal‐directed reward‐seeking behavior [123]. Of the dopamine D1R‐expressing direct pathway, and the dopamine D2 and the adenosine A2AR‐coexpressing indirect pathway MSNs, the activation of designer receptors exclusively activated by designer drugs (DREADDs), fabricated in nearby astrocytes, reduced the frequency of sEPSCs in the indirect pathway MSNs, but not in the direct pathway MSNs. This chemogenetic stimulation of astrocytes abolished habitual reward‐seeking behavior and promoted the transition to goal‐directed reward seeking behavior. Mice genetically lacking ENT1 did not show such a transition, confirming that the endogenous adenosine level participates in this effect. These series of experiments agreed with the finding that L‐α‐aminoadipate (a selective metabolic toxin of astrocytes), both under ex vivo and in vivo conditions, depressed LTP magnitude in the CA1 area of the hippocampus and in addition impaired hippocampal‐dependent memory (measured with the novel object recognition and object displacement tests) in mice [119].

The A2AR agonist CGS21680 facilitated adenosine uptake into hippocampal synaptosomes and also increased from these synaptosomes the depolarization‐induced release of adenosine [124]. By contrast, either the blockade of the ENT by nitrobenzylthioinosine or inhibition of the A2AR by SCH58261 abrogated these effects. These results indicated that A2AR activation facilitated the activity of the nucleoside transporters and its blockade inhibited the outflow of adenosine from the synaptosomal preparations. By using a genetically encoded G protein‐coupled receptor (GPCR)‐activation‐based adenosine fluorescent sensor (CRAB_Ado_), it was discovered that neuronal activity releases adenosine from the somatodendritic compartments rather than from the nerve terminals of hippocampal neurons [125]. It was also shown that adenosine release depends on ENT and requires calcium influx through L‐type Ca^2+^ channels. A similar ENT‐2‐dependent adenosine release was observed in the cerebral cortex after 40 Hz light flickering [126]. The resulting enhanced glymphatic flow was due to increased cerebrofluid adenosine levels activating astrocytic A2ARs, which themselves interacted with aquaporin‐4 channels of the same astrocytes. Glymphatic flow is fundamental for the homeostasis of the brain milieu eliminating metabolic waste. Since both inhibition of ENT‐2 and exclusion of A2ARs blocked the enhanced glymphatic flow, an astrocytic function appears to be involved, although the increased release of adenosine itself is due to a neuronal effect. In fact, cortical (glutamatergic and GABAergic) neurons, rather than astrocytes, were the cellular sources of adenosine outflow via the function of an ENT‐2 as the molecular pathway [127]. The increased ENT‐2‐mediated release of adenosine enhanced non‐rapid eye movement (non‐REM) and REM sleep in mice, which by itself enhanced cognitive functions.

A small adenosine analogue J4 blocks ENT‐1 and thereby prevented the decline in memory in the APP/PSI mouse model of AD [128]. Chronic treatment with J4 normalized LTP at CA3–CA1 synapses, as well as counteracted the aberrant expression of synaptic proteins, and the detrimental elevation in astrocytic A2AR expression in the hippocampus. Hence, the ENT1‐mediated release of adenosine during AD activated A2ARs, causing neurodegenerative damage to the hippocampus and cortex. Similarly, treatment with the ENT1 inhibitor J4 exerted beneficial effects in a mouse model of tauopathy [129]. Thus, energy dysfunction (including mitochondrial impairment) was improved, pathological astrocytic activation was prevented, and synaptic functions/memory processes were ameliorated.

Neuroinflammation is involved in cognitive deficits and neurodegenerative illnesses. Application of lipopolysaccharide (LPS) to ENT‐2 KO mice and their wild‐type controls showed an attenuation of the LPS effects in the KO mice [130]. Thus, ENT‐2 plays an important role in regulating inflammation‐associated cognitive decline and neural damage.

ENT‐1 and ENT‐2 were found both on neuronal and astrocytic plasma membranes [131] just as A2ARs. Therefore, the functioning of, for example, an A2AR‐modulated ENT‐1 effect is by no way a proof for the astrocytic regulation of adenosine release into the extracellular space. However, the opposite notion does not hold true either. In the present section, some but not all data suggest astrocytic involvement in the regulatory functions of ENT1/2, but in many cases, this affirmation is doubtful or even can be excluded with high certainty.

#### 4.6.2 Changes of Adenosine Levels Arising from the Degradation of Astrocytic ATP are Causes of Cognitive Deterioration

Further evidence also convincingly proved that in a β‐amyloid (Aβ_1–42_)‐based mouse model of early AD, an increased synaptic release of ATP coupled to an increased density and activity of CD73 is of causative significance for the cognitive deterioration [132]. CD73 was shown according to expectations to facilitate the formation of adenosine, which on its behalf selectively activated A2ARs. The enhanced CD73 activity was critically required for Aβ_1–42_ to impair synaptic plasticity (CA3–CA1–LTP in hippocampal slices) and memory, since the cognitive deficits were eliminated in A2AR KO mice as well as forebrain A2AR and CD73 KO mice. A2ARs were shown to control fear memory and the underlying processes of synaptic plasticity in the amygdala [133]. The repeated bilateral cerebroventricular injection of the CD73 inhibitor α,β‐methylene ATP (α,β‐meATP) imitated the effects of selective A2AR blockade by SCH58261 both on fear memory and amygdala‐LTP, proving that the enzymatic degradation of released ATP to adenosine is the initiator of these effects. Similarly, the ATP‐derived formation of extracellular adenosine bolstering A2AR activation was identified as a key pathway responsible for abnormal synaptic plasticity in circuits involved in the onset of PD motor symptoms [134].

Neuroligins, a family of cell adhesion molecules, are essential for synapse development and their dysfunction is linked to social memory disturbances (measured by three‐chamber sociability and social novelty tests) via A2AR downregulation [135]. These data were generated in adult male mice with the astrocytic deletion of neuroligin‐3 in the ventral hippocampus.

### 4.7 Involvement of A1 and A2A/A2B Receptors in Cognitive Deterioration

A1Rs are coupled via G_i,o_ proteins to adenylate cyclase and their activation decreases the cAMP concentration and thereby the tonic activity of protein kinase A (PKA) [133, 134, 135, 136]. They depress neuronal excitability by a mixed pre‐ and postsynaptic effect; presynaptic A1Rs located at glutamatergic neuronal terminals decrease the release of the excitatory glutamate and postsynaptic A1Rs open inwardly rectifying potassium channels thereby causing hyperpolarization. A2ARs are coupled via G_s_ proteins to adenylate cyclase, and their activation increases cAMP concentration and thereby the stimulation of PKA in the striatum; presynaptic A2ARs located at glutamatergic nerve terminals increase the release of the excitotoxic glutamate and postsynaptically they lead to the opening of voltage‐sensitive Ca^2+^ channels. Whereas A1Rs have a widespread distribution in the CNS, A2ARs are preferentially expressed in the striatum, although they can be found also in the hippocampus and cerebral cortex albeit at lower densities.

However, outside of the striatum, the behaviorally relevant signaling mechanisms of postsynaptic A2ARs (located at synapses between mossy fibers and CA3 pyramidal neurons) are independent from PKA stimulation. In the hippocampus, they spared mossy fiber stimulation‐dependent AMPA–EPSCs, but potentiated NMDA–EPSCs involved in the induction of LTP in CA3 cells [136]. Thus, postsynaptic A2ARs might affect information processing in CA3 neuronal networks and possibly also memory performance.

The inhibition of gliotransmission in dnSNARE mice attenuated the accumulation of sleep pressure, assessed by measuring the slow wave activity of the EEG during non‐REM sleep, and also prevented the cognitive deficits (measured by the novel object recognition test) associated with sleep loss [11, 137]. Since the sleep‐suppressing effects of the A1R antagonist 8‐cyclopentyl‐1,3‐dimethylxanthine (CPT) was prevented following gliotransmission inhibition, and because intracerebroventricular application of CPT to wild‐type mice mimicked the transgenic phenotype, it was concluded that A1Rs were involved in the reported effects of sleep‐deprivation.

The specific optogenetic stimulation of astrocytes in glial fibrillary acidic protein–channelrhodopsin 2 (*ChR2*)‐enhanced yellow fluorescent protein rats disrupted memory consolidation of fear‐related anxiety behavior [138]. Intracerebral blockade of A1Rs reversed the attenuation of fear memory, while intracerebral injection of an A1R agonist, 2‐chloro‐N^6^‐cyclopentyladenosine (CCPA) mimicked the effect of astrocyte activation. Apparently, optogenetic stimulation caused adenosine release from astrocytes, which then activated A1Rs and interfered with the consolidation of anxiety behavior.

High frequency stimulation of the cortical inputs induced LTD, mediated by A1R activation at corticostriatal synapses of the direct pathway in the dorsolateral striatum [139]. It was found that cortical high frequency stimulation increased calcium levels in striatal astrocytes through activation of mGluR5‐R signaling and that this astrocyte‐mediated response is necessary for A1R‐mediated LTD. In agreement with this finding when G_q_ receptors were chemogenetically activated, A1R‐mediated synaptic depression evolved at cortico‐dSPN synapses.

Caffeine is the most consumed psychostimulant in the world and is known to affect basic and fundamental neuronal processes such as sleep, arousal, cognition as well as learning and memory [140]. These effects are exclusively due to the blockade of A1 and A2ARs (and not of other actions, e.g., release of intracellular Ca^2+^, elicited by toxic concentrations of caffeine only), although the modulation of synaptic plasticity, and the beneficial effects of caffeine in the neurogenerative illnesses AD and PD are caused by A2AR inhibition [48, 49, 132, 141, 142].

A2ARs are potential candidates to modulate bidirectional communication between neurons and astrocytes, thus shaping synaptic plasticity, which underlies learning and memory [143]. The mixed A1/A2AR antagonist caffeine was found to decrease in hippocampal slices the CA3–CA1–LTP amplitude, an effect mimicked in A2AR KO mice or by pharmacological blockade of A2ARs by SCH58261 [141]. These findings suggest that endogenous adenosine acting at A2ARs potentiates LTP under control conditions, although there is no information with this experimental approach on the astrocytic or neuronal localization of A2ARs [141]. However, genetic silencing of A2ARs selectively in hippocampal astrocytes alters astrocytic morphology and leads to deficits of spatial reference memory (Y maze test), and compromises hippocampal synaptic plasticity, typified by a reduction of LTP magnitude and a shift of LTD towards LTP [144].

In another study, selective knockout of A2ARs in mouse astrocytes altered glutamate homeostasis by causing aberrant glutamate transporter‐1 (GLT‐1) activity [145]. The Na^+^‐dependent GLT‐1 controls the uptake of glutamate preferentially into astrocytes. The dysregulation of GLT‐1 in consequence of the missing regulation by A2ARs led to a decrease of working memory. In good agreement with the regulation of astrocytic functions by A2ARs, the overexpression of GLT‐1 promoted robust transcriptional changes, mostly affecting immune responses, angiogenesis and cell activation‐related genes in a primary astrocytic culture [146]. Investigations of other authors also showed that altering astrocytic function with either glial toxins [119, 147], or different genetic [148, 149] or chemogenetic manipulations [74, 150] result in impaired spatial memory. Hence, both astrocytic and neuronal A2ARs may modulate hippocampal synaptic plasticity and memory, albeit with different consequences.

The dorsal and ventral hippocampal circuits both control mood via differential A1/A2AR‐mediated modulatory mechanisms [151, 152, 153]. Nonetheless, these effects have not been reported to depend on the astrocytic release of adenosine.

Until now, we discussed the role of astrocytic A1/A2ARs in the regulation of cognitive performance because of their activation by low concentrations of adenosine; nonetheless, A2BRs, much less sensitive to adenosine, also appear to execute important functions [154]. Stimulation of A2BRs recruits a cAMP–PKA signaling pathway, resulting in rapid activation of astrocyte glucose metabolism and the release of lactate, which supplements the extracellular pool of readily available energy substrates. These data identify the A2BRs of astrocytes as sensors of neuronal activity, which results in the enhanced release of ATP/adenosine tuning brain metabolism.

### 4.8 Participation of A2A Receptors in AD

As already mentioned in Section 4.4, and further discussed in Section 4.6, AD is the most common form of dementia in the elderly, and is characterized by a deterioration of memory and other cognitive functions [155]. Aging, already independent of AD, differentially modulates A1 and A2AR‐dependent synaptic plasticity in three age groups of rats [156]. The selective A2AR antagonist SCH58261 attenuated hippocampal CA3–CA1–LTP, with the largest effect in the aged in comparison with two younger groups of rats. By contrast, the selective A1R antagonist DPCPX increased the LTP magnitude in young adult rats, without an effect in the other age groups. In agreement with these findings, the A2AR–mRNA expression was the highest in the hippocampus of aged rats out of the three groups of animals. Similarly, a significant overexpression of A2ARs was reported in hippocampal neurons of aged humans, which was aggravated in AD patients [117]. Hence, the upregulation of A2ARs appeared to be sufficient to drive memory impairment observed in old and AD‐afflicted humans.

Accordingly, it was found that the antagonism of A2ARs can recover memory deficits in animal models of AD. In rats, injected with Aβ_1–42_ via the intracerebroventricular route, both caffeine and the selective A2AR antagonist SCH58261 prevented synaptotoxicity and consequent cognitive impairment [155, 157, 158]. This was suggested to be associated with the increased levels of G_s_‐coupled A2ARs in astrocytes [150]. Conditional genetic removal of astrocytic A2ARs enhanced long‐term memory in young and aging mice, while chemogenetic activation of astrocytic G_s_‐coupled signaling reduced long‐term memory.

A number of astrocyte‐dependent possible mechanisms were identified through which A2ARs might perform impairment of cognitive functions in AD. First, in astrocytic primary cultures prepared from rat cortices and exposed to Aβ_1–42_, ATP was released through Cx43 hemichannels, in a manner blocked by the A2AR antagonist SCH58261 and mimicked by an A2AR agonist CGS21680 [159]. Hence, Aβ_1–42_ triggered ATP release through Cx43 hemichannels, a process blocked by A2AR antagonists and mimicked by A2AR agonists. A2ARs directly regulated hemichannel activity and prevented Cx43 upregulation observed in Aβ_1–42_‐exposed astrocytes. Second, it was confirmed that APP/PS1 AD‐model mice display deficits in hippocampal‐dependent memory (measured by the Morris water maze test), an accumulation of Aβ plaques and an increased astrocyte arbor complexity in the hippocampus [144]. In addition, enhanced activity of astrocytic Cx43 hemichannels was also observed in the hippocampus of these mice. The pharmacological blockade or genetic silencing (both global and astrocyte‐specific) of A2ARs prevented Aβ_1–42_‐induced hemichannel dysregulation in hippocampal slices. Third, in primary cultures of rat astrocytes exposed to Aβ_1–42_, ATP evoked Ca^2+^ responses had a lower amplitude but a longer duration than in Aβ_1–42_‐untreated astrocytes and involved P2X7 and P2Y1R activities [160]. The A2AR antagonist SCH58261 regulated both P2×7 and P2Y1R‐mediated [Ca^2+^]_i_ responses in astrocytes, confirming that A2ARs controlled the P2X7 and P2Y1R‐mediated [Ca^2+^]_i_ dynamics, which are disrupted in conditions of early AD.

## 5 Purinergic Causes of Affective Diseases

### 5.1 Major Depressive and Bipolar Disorders and their Rodent Models

MDD is a mental illness with symptoms of extreme sadness, depressed mood, and loss of interest that persists for at least 2 weeks and interferes with the social functioning and working ability of the afflicted individuals [54, 102, 161]. Bipolar disorder is characterized by alternating depressive and manic episodes; the latter periods are defined by increased psychomotor activity and elevated self‐esteem. Whereas the lifetime prevalence of MDD amounts to 15–18% in the general population [162, 163], that of bipolar disorder is only about 3% [164]. Both diseases are thought to be caused by the interaction of genetic, environmental, psychological and social factors. Especially long‐lasting stress exposure is considered to be the main pathogenetic factor of MDD. Hence, routinely used rodent models of MDD are based on the delivery of acute (tail suspension test, TST; forced swim test, FST; foot shock combined with sucrose preference test, SPT) or chronic (chronic unpredictable mild stress, CUMS; chronic social defeat stress, CSDS) stressful stimuli [165, 166], although it is absolutely clear that acute in contrast to chronic stress does not lead to abnormal adaptive behaviors typical for MDD. The consequence of TST and FST is “behavioral despair,” manifesting itself in mice suspended by their tails or swimming in water, in prolongation of immobility due to giving up futile escape reactions. The classic rodent model of mania consists of the systemic application of amphetamine or ouabain and the measurement of increased locomotion [167].

### 5.2 Impeded Exocytotic ATP Release from Astrocytes in the Hippocampus and Medial (m)PFC are Causes of Depressive‐Like Behavior

**FIGURE 5:**
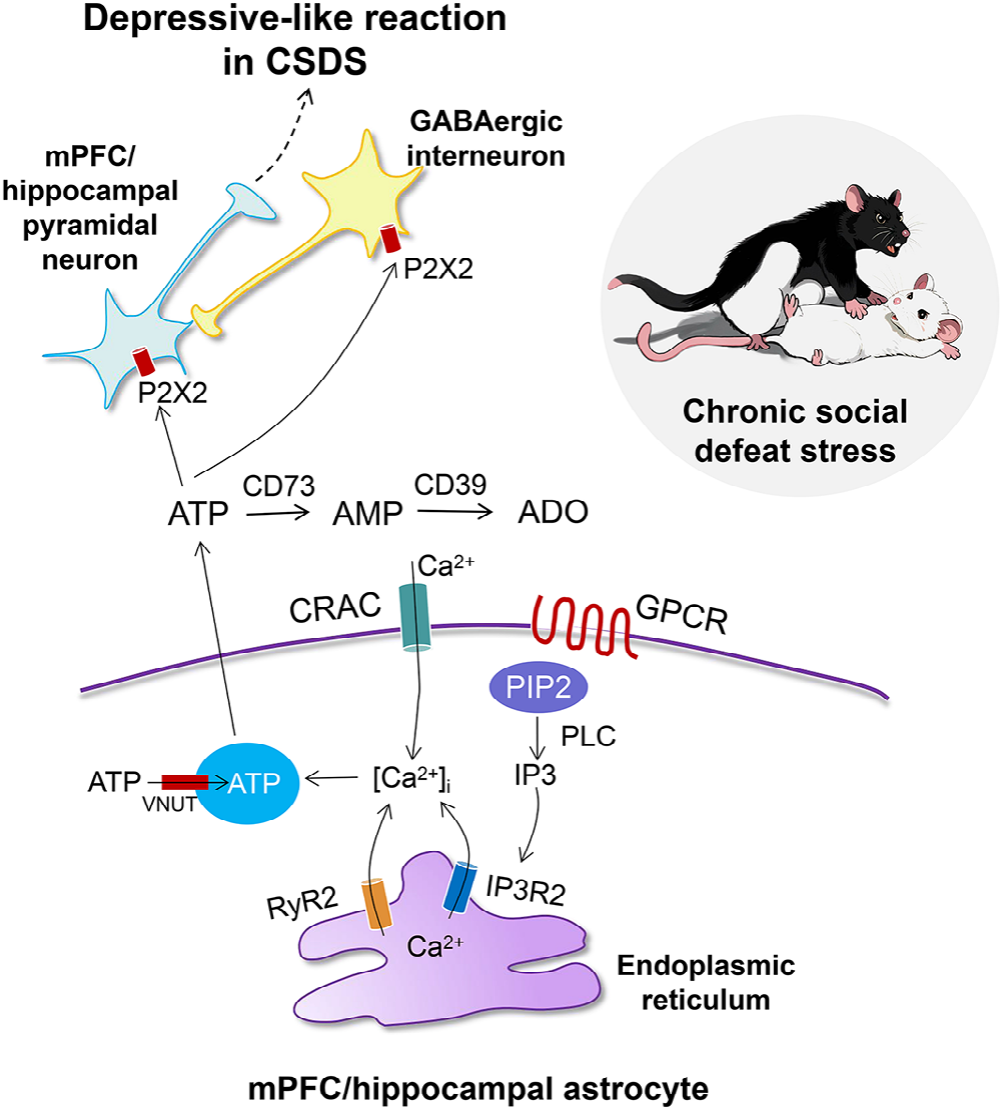
Impeded exocytotic ATP release from astrocytes in the hippocampus and medial prefrontal cortex is one of the causes of depressive‐like behavior. A plethora of experimental findings suggests that in the chronic social defeat stress (CSDS) test a low extracellular ATP concentration in the medial prefrontal cortex (mPFC) and hippocampus mediates depressive‐like behavior. Vesicular ATP release from astrocytes is induced by an elevation of [Ca^2+^]_i_. One of the reasons may be the activation of a transmembrane G protein‐coupled receptor (GPCR) or under experimental conditions the activation of a G_q_‐coupled artificial receptor by designer receptors exclusively activated by designer drug (DREADD); during this procedure an antidepressant effect developed in the CSDS test. Similarly, knockout of the IP3R2, or the ryanodine receptor‐2 (RyR2) both normally stimulating Ca^2+^ release from the endoplasmic reticulum, causes depressive‐like behavior. The genetic exclusion of calcium release‐activated calcium channel protein 1 (CRAC1) leads to the amelioration of depressive‐like behavior. Blockade of the vesicular nucleotide transporter (VNUT) or the inactivation of extracellular ATP by the stimulation of the ectonucleotidase CD39 also bring about the depressive‐like behavior. Eventually, conditional knockout of P2X2Rs located at mPFC (hippocampal) pyramidal neurons or GABAergic interneurons innervating them initiates depressive‐like behaviors, and the overexpression of these receptors has the opposite effect. PIP2, phosphatidylinositol 4,5‐bisphosphate; PLC, phospholipase C; CD73, 5′‐nucleotidase.

In the last couple of years, experimental work on rodent models has identified the gliotransmitter ATP as one of the primary executors for the development of depressive‐like behavior [168, 169, 170]. Both decreased and increased ATP release is thought to be involved in the etiology of MDD; thus, the role of extracellular ATP by itself (or after degradation to adenosine) in the control of mood is multifaceted (see Sections 5.2–5.5). Furthermore, extracellular ATP concentrations in the brain of mice, that were susceptible to CSDS, were permanently low [11] (Figure 5). Blockade of vesicular ATP release from astrocytes either by knockout of IP3R2 or by generation of a transgenic dnSNARE mouse led to depressive‐like reactions that could be rescued via the i.p. application of ATP. Moreover, the selective stimulation of ATP release from astrocytes of transgenic mice that expressed a G_q_ protein‐coupled artificial receptor, induced antidepressant‐like effects. Hypostimulation of P2X2Rs in the mPFC was supposed to be causally related to depressive‐like behavior. In a follower paper of the same group of authors, the target of astrocyte‐derived ATP was identified at P2X2Rs of mPFC pyramidal neurons [164]. Conditional knockout of P2X2Rs in these pyramidal neurons promoted resilience to chronic stress‐induced depressive‐like behaviors, and specific gain of P2X2Rs by their overexpression with adeno‐associated virus (AAV)‐P2X2 increased the vulnerability to such behaviors.

In perfect agreement with the above data, the blockade of the enzyme CD39 (ENTPD), which hydrolyses extracellular ATP, counteracted the degree of depressive‐like behavior in the CSDS test [171]. Thus, increased ATP levels, in this case in the hippocampus by direct effect (but not by the indirect one through adenosine, see Section 5.5), beneficially modified the CSDS‐induced depressive‐like behavior of mice. Eventually, ex vivo slice electrophysiology documented that the observed extracellular ATP deficiency both in CSDS and IP3R2 KO mice reduced GABAergic inhibition and elevated excitability in lateral habenula‐projecting, but not dorsal raphe‐projecting mPFC neurons [172]. In contrast to previous results, it was found that GABAergic interneurons rather than pyramidal neurons were endowed with P2X2Rs and mediated ATPergic modulation of depressive‐like behavior.

Classic antidepressive therapy with, for example, the selective 5‐HT reuptake inhibitor fluoxetine might also act, at least partially, via increasing the pathologically low astrocytic ATP release [173]. ATP exocytosis relies on the unimpaired functioning of the VNUT. Fluoxetine‐induced antidepressive‐like behavior was decreased in mice, whose astrocyte‐selective VNUT was genetically deleted, but was increased when astrocyte‐selective VNUT was overexpressed. These findings suggest that in addition to neurons, fluoxetine acts on astrocytes and can mediate its therapeutic effect by increasing ATP gliotransmission. However, it has to be noted that of the routinely used classes of antidepressants, the tricyclic imipramine, but not the selective 5‐HT reuptake inhibitor fluoxetine might exert an antidepressive‐like effect by modulating astrogliogenesis in the dentate gyrus of rats exposed to CUMS [174].

Exocytotic gliotransmitter release may be due to Ca^2+^ influx triggered by ORAI1‐3 channels. The calcium release‐activated calcium channel protein 1 (CRAC1) is formed by the ORAI1‐3 proteins and serves in non‐neuronal cells store‐operated Ca^2+^ entry [175]. The depletion of endoplasmic reticulum Ca^2+^ stores activates ORAI1‐3 channels in the plasma membrane and thereby results in the replenishment of the intracellular Ca^2+^ stores. Knockout of this type of transmembrane channels has been reported to show amelioration of LPS‐induced depression‐like behaviors including learned helplessness and anhedonia [176] (for the participation of neuroinflammatory signaling in such behaviors see Section 5.4).

In clinical praxis, individuals with MDD frequently experience symptoms of anxiety or have comorbid anxiety disorders [177]. In this context it was interesting to learn that optogenetic hippocampal astrocyte activation elevating intracellular calcium, induced anxiolytic behavior in astrocyte‐specific ChR2 transgenic mice [178]. ATP released from the activated hippocampal astrocytes increased excitatory synaptic transmission in dentate gyrus granule cells, which exerted anxiolytic effects.

### 5.3 Impeded Channel‐Mediated ATP Release from Astrocytes in the Hippocampus and mPFC is a Cause of Depressive‐Like Behavior

Ample evidence indicates that blockade of gap junctions and hemichannels induces depressive‐like behavior in rodent models of MDD [179, 180]. For example, the expression of Cx30 and Cx43 was found to be significantly decreased in the mPFC and hippocampus of CSDS mice and was strongly associated with decreases in neuronal activity as measured by electrophysiological methods in slices prepared from these areas of the brain [181]. Moreover, overexpression of Cx30 and Cx43 in the mPFC and hippocampus increased neuronal activity and inhibited depressive‐like behavior. The antimalarial drug mefloquine has frequent side‐effects, such as depression and anxiety, and is known as an inhibitor of Panx‐1 channels. In the CSDS mouse model of depressive‐like behavior a decrease in the expression and function of Panx‐1 channels has been observed in the mPFC of susceptible mice [182]. Pharmacological blockade of Panx‐1 by carbenoxolone induced depressive‐like behavior, which was prevented by preconditioning with ATP. Systemic and intra‐mPFC injection of mefloquine inhibited the activity of Panx‐1 and induced depressive‐like and anxiety behaviors in mice.

As quite often, also in this case, there are two opposite results available in the literature. Dye uptake experiments in hippocampal slices revealed that acute restraint stress induces the opening of both Cx43 hemichannels and Panx‐1 channels in astrocytes, which were further increased by chronic restraint stress [183]. Blockade of these channels reduced ATP and glutamate release from hippocampal slices of stressed mice. Why channel closure in one case and opening in the other induces depressive‐like behavior is unclear, although it has to be mentioned that methodological differences (e.g., dye uptake versus electrophysiology) might be one of the explanatory factors.

In addition to connexin hemichannels and pannexin channels, other type of transmembrane channels may function in astrocytes as ATP secretory pathways. Such are the CALHM2 channels, which can directly mediate ATP release. Conventional knockout and conditional astrocyte‐specific knockout of CALHM2 both led to significantly reduced ATP concentrations, loss of hippocampal spine number, and depression‐like behaviors in mice [184]. All these reactions can be rescued by systemic ATP replenishment. It has been previously reported that inescapable foot shock caused an acute and persistent loss of spine synapses in all hippocampal subfields (CA1, CA3, dentate gyrus), which was associated with escape deficit in learned helplessness [185]. The analysis of single nucleotide polymorphism showed that the CALHM2 V136G small nucleotide polymorphism (SNP) is significantly associated with the ATP‐release function of astrocytes and results in depressive‐like behavior that is rescued by application of exogenous ATP [186].

### 5.4 Upregulation of P2X7 Receptor During Neuroinflammation is a Cause of Depressive‐ or Mania‐Like Behavior

In extension of the previously reported data, we also refer herewith to results proving increased ATP release and the emergence of the P2X7R under neuro‐inflammatory conditions, which is associated with stress‐evoked depressive‐like reactions.

Acute restraint stress rapidly increased the levels of extracellular ATP, inflammatory cytokines, and the active form of the NLRP3 inflammasome in the hippocampus of rats or mice [187]. Administration of P2X7R antagonists blocked the release of these cytokines and reversed also the anhedonic and anxiety behaviors caused by CUMS exposure. Moreover, deletion of the *Nlrp3* gene, coding for the NLRP3 inflammasome rendered mice resistant to the development of depressive‐like behaviors caused by CUMS. Similar effects were observed also by another group of authors, utilizing again chronic rodent models of MDD; they concluded that P2X7Rs located at hippocampal microglia rather than astrocytes mediate the depressive‐like reactions [188]. Systemic injection of LPS initiated in mice an increase of the serum concentration of TNF‐α, and increased the immobility time in TST and FST. Both the cytokine increase in the serum and the depressive‐like responses were abrogated by the application of Brilliant Blue G, a selective P2X7R antagonist [189]. However, this experiment did not differentiate between microglia or astrocytes as sources of cytokine secretion.

P2X7Rs and the NLRP3 inflammatory pathway leading to neuroinflammation and in consequence major depression (depressive‐like behavior) appears to be initiated not only by the activation of microglia, but most probably also by activated astrocytes [190, 191]. In fact, in a rodent model of MDD, in which chronic sleep deprivation induced depressive‐like reactions, astrocytic, in contrast to microglial P2X7Rs turned out to be the major etiological factors [192]. Similarly, TST, FST, and SPT (after foot shock) determinations have shown in rodent models, the acquirement of P2×7R‐medited behavioral despair and anhedonia reactions, respectively [161]. It was concluded by the use of drugs preferentially interfering with the function/metabolism/mitotic activity of microglia (minocycline), astrocytes (L‐α‐aminoadipate), and oligodendrocytes (cytosine‐β‐arabinoside), that pharmacological damage to microglia and astrocytes causes blockade of all types of acute depressive‐like reactions, while injury to astrocytes inhibits only reactions induced by strong stressors, such as foot shock. When the expression profiles of mRNAs for Cx43, P2X7Rs, and 5’‐nucleotidase were examined in cerebro‐cortical and hippocampal astrocytes (identified by magnetic cell sorting), increased levels of these cellular constituents were noticed [193]. Thus, unequivocal evidence confirms the upregulation and behavioral involvement of P2X7Rs in rodents subsequent to acute or chronic stressful stimulation.

It is interesting to mention that brain‐region specific alterations of epoxyeicosatrienoic acid (EET) signaling, which is an arachidonic acid metabolic pathway, was observed both in a mouse model of MDD (CSDS) and postmortem samples from patients with this disorder [194]. The enzymatic activity of soluble epoxide hydrolase (sHE), the key enzyme in EET signaling was selectively increased in the mPFC of susceptible mice after the application of CSDS, in an ATP release‐dependent manner. Actually, sHE was primarily expressed in the lysosomes of astrocytes suggesting their involvement in vesicular ATP secretion. Accordingly; higher expression of sHE protein was found in the postmortem brain samples of patients with depression [195]. A study in women with gestational diabetes mellitus found association between depressive symptoms and several SNPs of epoxide hydrolase‐2 encoding sHE [196].

Astrocytes highly express the VNUT, which takes up ATP into storage vesicles or secretory lysosomes of neurons/astrocytes [197]. Another ω‐3‐polyunsaturated fatty acid, eicosapentaenoic acid (EPA) has been shown to inhibit VNUT, thereby impairing vesicular ATP release from neurons, without affecting the vesicular release of other neurotransmitters. EPA potently attenuated neuropathic and inflammatory pain in wild‐type mice but not in VNUT^−/−^ mice [198]. In addition, ω‐3‐polyunsaturated fatty acids, especially EPA have been shown to have an overall beneficial effect on depression symptoms as reported for humans [199]. Hence, we tentatively suggest that the improvement by EET in CSDS‐induced depressive‐like behavior depends on the inhibition of the vesicular ATP release.

In contrast to MDD and depressive‐like behavior, only few animal studies dealt with the participation of P2X7Rs in the rodent, amphetamine‐model of mania, although they were without exception affirmative [200, 201, 202, 203]. However, none of these studies indicated that astrocytic rather than neuronal P2X7Rs are the targets of endogenous ATP.

### 5.5 Changes in Adenosine Levels Arising from the Degradation of Astrocytic ATP are Causes of Depressive‐Like Behavior

The enzymatic degradation product of ATP, adenosine has also been reported to be involved in the induction of depressive‐like behavior. In agreement with the finding that purinergic signaling orchestrates neuron‐glia communication [96, 204], modulation of adenosine synthesis, transport, catabolism, and receptors, all affected responses to acute or chronic stress in rodent models of MDD [205, 206, 207, 208]. However, in these cases there was no indication for the astrocytic release of ATP identified as a source of adenosine or the astrocytic location of, for example, A1Rs in question [209, 210, 211]. By contrast, the systemic administration of the selective A1R antagonist 8‐cyclopentyl‐1,3‐dipropylxanthine (DPCPX) counteracted the antidepressant activity of the NMDA‐R antagonistic ketamine in the mouse FST by a purely neuronal mechanism [212].

**FIGURE 6:**
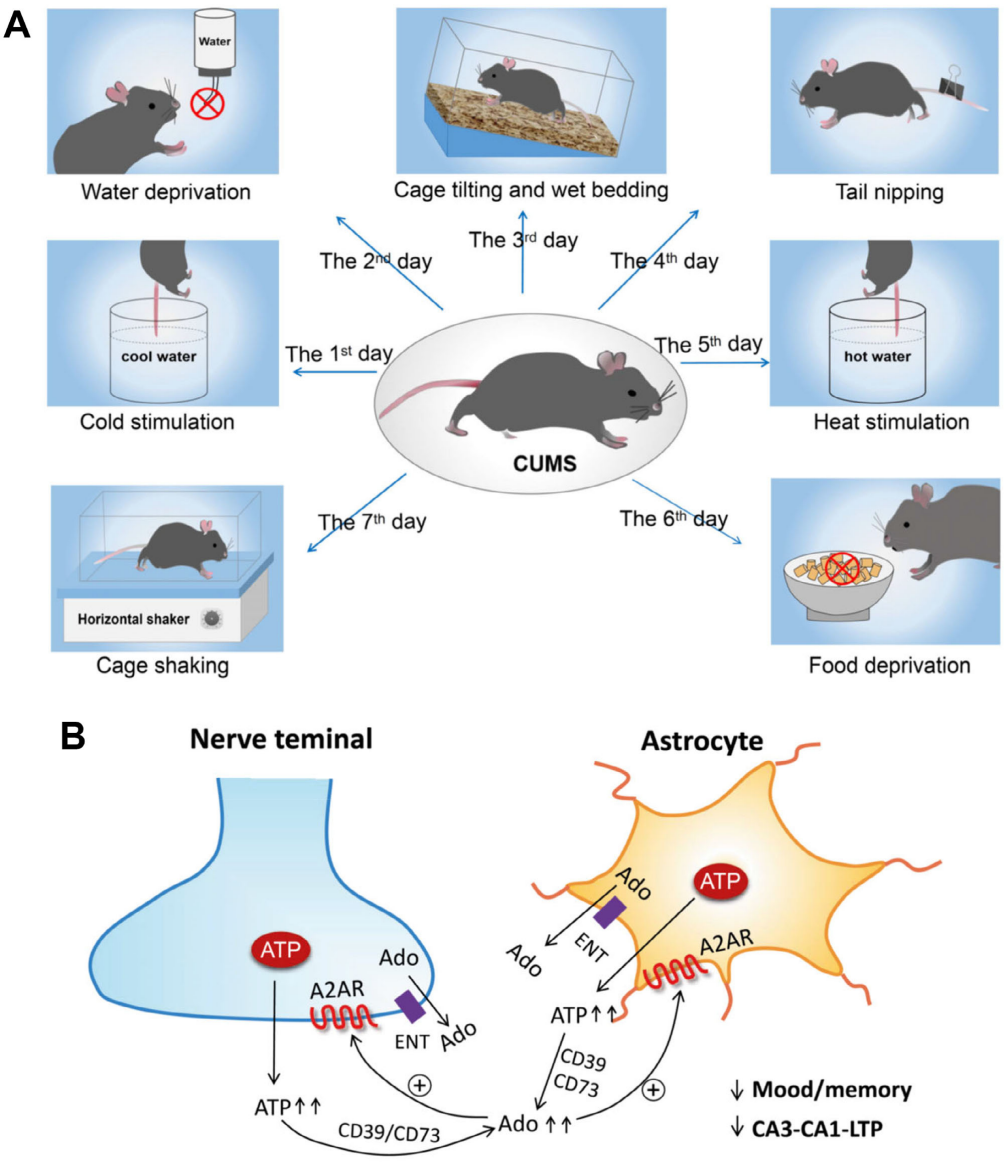
Facilitated exocytotic ATP release from neurons and probably also astrocytes in the hippocampus and medial prefrontal cortex is one of the causes of depressive‐like behavior; an increased transporter‐mediated outflow of adenosine from neurons and astrocytes may also add up to the development of this state. (A) Chronic unpredictable mild stress (CUMS) leads to the evolution of a depressive‐like state [214]. CUMS is a procedure in which usually for the duration of a month rodents undergo mild stressful stimulations (e.g., cold stimulation of their tails, water deprivation, cage tilting and wet bedding, etc.) for a certain period of time each day. (B) This leads to boosted accumulation of adenosine in the extracellular space, (1) either because of increased exocytotic ATP release from nerve terminals with the consequent enzymatic degradation of this ATP to adenosine [160], or by further pathways which might also participate in the elevation of extracellular adenosine such as (2) exocytotic release of ATP from astrocytes, (3) outward transport of adenosine by the equilibrative adenosine transporter (ENT) from neurons, (4) a similar effect of ENT from astrocytes. Increased stimulation of A2ARs at neurons and astrocytes is supposed to downregulate mood/memory and the associated CA3–CA1–LTP in the hippocampus and the layer II/III‐layer V LTP in the medial prefrontal cortex. The possibilities summarized under the paragraphs (2–4) are hypothetical but represent subjects for future research.

Peripheral injection of LPS to mice evoked systemic inflammation, and rapidly increased the plasma levels of adenosine, triggering astrocyte reactivity via A1R activation [213]. The stimulation of A1Rs on their behalf increased the levels of inflammatory factors, thereby alleviating microglial reactions, causing among others depression‐like behavior. A1R deficiency in astrocytes inhibited these effects, while chemogenetic stimulation of G_i_ protein signaling restored neuroinflammation and depressive‐like behavior. Although the astrocytic source of ATP was not proven either, a particularly impactful study still deserves mentioning [160]. In chronically restraint stressed rats, an increased release of ATP, its extracellular catabolism through CD73 to adenosine, and the subsequent overactivation of A2ARs in the frontocortical and hippocampal regions of rats was demonstrated [160]. Synaptosomes prepared from the mentioned areas of restrain stressed rats exhibited increased ATP release on depolarization by elevated extracellular potassium concentration. The continuous intracerebroventricular delivery of the CD73 inhibitor α,βme‐ATP during the restraint stress procedure attenuated mood and memory dysfunction and also prevented the otherwise decreased LTP in the prefrontocortical layer II/III‐layer V, as well the hippocampal CA3–CA1 synapses. These findings are of great interest because of two reasons: (1) an increased rather than a decreased ATP release underlies the stress‐induced depression‐like reactions; and (2) the CD73‐produced adenosine acting via A2ARs is the cause of the interference with normal mood and cognitive behavior (Figure 6; see also [208]).

Stress‐induced depressive‐like reactions in mice TST and chronic restraint stress appear to be mediated by GABAergic A2AR‐containing neurons localized in the lateral septum region of the brain, via projections sent to the dorsomedial hypothalamus and the lateral habenula [215]. However, A2ARs might be expressed both by astrocytes and microglia in the CA1 area of the ventral hippocampus participating in the anxiodepressive consequences of cheek pain. The astrocytic metabolic inhibitor fluorocitrate, the CD39 (ENTPD) inhibitor ARL 67156 and A2AR blockade attenuated the increases of extracellular ATP and adenosine, and the consequent pain‐related anxiodepression [216].

## 6 P2X7 Receptor‐Related Diagnostic and Therapeutic Maneuvers

### 6.1 Diagnostic Use of Blood Plasma or Serum Determination of P2X7 Receptors

A wealth of publications reports that P2X7Rs participate in various neurodegenerative diseases in general [217, 218, 219] and AD in particular [58, 79, 220]. All these articles unequivocally conclude that such CNS illnesses lead to increased levels of P2X7Rs accompanying neuroinflammation, inevitably superimposed on the core causative factor (usually the extracellular deposits of pathological protein aggregates).

It was shown as early as 2006 that stimulation of the P2X7R by its prototypic agonist dibenzoyl‐ATP (Bz‐ATP) in dendritic cells (DCs; professional antigen‐presenting cells initiating the immune response) kept in culture led to fast microvesicle shedding from the DC plasma membrane [221]. These microvesicles contained P2X7Rs, major histocompatibility complex II, and CD39. Based on that, two parallel studies identified in blood plasma/serum of temporal lobe epileptic (TLE; [222]) or COVID‐19 [223] patients, the increased level of soluble or shed (s) trimeric/monomeric P2X7Rs [224]. Soon afterward it was reported that AD patients also exhibit higher plasma concentrations of sP2X7R [225]. Hence, in CNS diseases (TLE, AD) and mixed central and peripheral illnesses (COVID‐19) a uniform increase of the (neuro)inflammatory marker P2X7R was verified. Independent of these experiments, the blood levels of sP2X7Rs were shown to accord with the classic inflammatory marker C reactive protein [226]. Further, in this latter study the sP2X7R in the plasma was largely associated with microvesicles. Sustained activation of P2X7Rs apparently triggered the release of matrix metalloproteinase 2‐dependent receptor cleavage [227]. Thus, the sP2X7R might be a full receptor protein, its single subunit, or only the ectodomain of the receptor.

### 6.2 Radioligand Targeting of P2X7Receptors

The in vivo imaging techniques positron emission tomography (PET) and single photon emission computed tomography (SPECT) are methods with high sensitivity for diagnosis and treatment‐monitoring of P2X7R‐related diseases [228, 229]. PET surmounts SPECT in its sensitivity, and has usually used to recognize a 18 kDa translocator protein (TSPO) as a marker for inflammatory changes of the microglial activation state [230]. However, TSPO had high inter‐subject variability in binding affinity, and nonspecific binding in the human brain due to TSPO polymorphism. This has necessitated the need to look for an alternative neuroinflammatory marker, which was found in the P2X7R [231]. Tritium (^3^H), carbon (^11^C), fluorine (^18^F), and iodine (^123^J) were used for labelling radioligands targeting P2X7Rs [228]. These radioligands were allosteric rather than orthosteric antagonists, with only [^11^C]GSK1482160, [^18^F]JNJ‐64413739, and [^11^C]SMW139 used in human studies [232, 233, 234]. The simultaneous application of shed P2X7 (sP2X7R) determination and the PET measurement of this receptor has a higher predictive value than that of only one of the methods.

### 6.3 P2X7 Receptors as Therapeutic Targets

We mentioned already previously that P2X7Rs are likely therapeutic targets for neurodegenerative diseases causing cognitive impairment and/or affective disturbances in animal models of these diseases [103, 235, 236]. P2X7R antagonists block the release of proinflammatory cytokines from macrophages and have been intensively investigated in peripheral autoimmune illnesses such as rheumatoid arthritis [237, 238] and Crohn's disease [239]. Nonetheless, these clinical studies were considered to be unsatisfactory, and were terminated [227, 240], although in moderate to severe Crohn's disease a favorable risk profile was observed [239]. Diverse pharmaceutical companies still continued their research activities for a couple of indications (rheumatoid arthritis, neuropathic pain, age‐related retinal degeneration, diabetic retinopathy, glaucoma), but again without the expected success [224]. However, in a double‐blind, placebo controlled, randomized clinical study, the blood–brain‐permeable P2X7R antagonist, JNJ‐54175446 albeit having no significant effects on mood perturbed by total sleep deprivation, reduced anhedonia caused by this manipulation [241].

Hence, the development of therapeutic agents to cure either central or peripheral inflammatory diseases, turned out to be of questionable value, although antagonists with high blood–brain barrier (BBB) permeability, and excellent peroral adsorption have been used for CNS therapeutic indications [242, 243]. Alternative approaches, with nanobodies (heavy chain antibodies) exhibiting high selectivity for P2X7Rs were not superior to the small molecular pharmacological antagonists, because in this case both the poor enteral adsorption and BBB permeability were serious hindrances [244, 245].

In spite of these mostly negative experiences with P2X7R antagonists, there is still room for further clinical studies, especially for the applicability in the case of CNS illnesses, such as cognitive and mood disorders, where experiments with rodents promise considerable beneficial effect. Unfortunately, AD and MDD investigations are most tedious, because the respective diseases are of chronic nature and a treatment has to be carried out over a long period of time.

Subgroups of patients, which are resistant to classic therapeutic approaches, might still respond to combinations of P2X7R antagonists with the presently known antidepressive or cognition enhancing drugs. Eventually, alternative therapeutic maneuvers in humans would be either the selective blockade of ATP release from microglia/astrocytes, or an interference with the multiple transduction mechanisms triggered by P2X7R activation. One of these latter possibilities would be the blockade of the inflammasome activation, which is an emerging field to combat neurodegenerative diseases. With respect to the blockade of the release of ATP and other gliotransmitters from astrocytes, metabolic toxins (L‐α‐aminoadipate, fluorocitric acid) were repeatedly used in animal experimentation, raising the hope that nontoxic substances with similar effects might also be developed for clinical praxis.

### 6.4 A2A Receptors as Therapeutic Targets

The A2AR appears to be involved in the pathophysiology of AD as already discussed in detail in Section 4.8. Approved drugs for AD primarily increase acetylcholine transmission and reduce glutamate excitotoxicity (donezepil, rivastigmine, galantamine, memantine [246, 247]). Moreover, recently a novel antibody decreasing extraneuronal Aβ protein accumulation in the brain, called aducanumab, has been approved by the US Food and Drug Administration (US FDA; [248]). However, with the first classes of drugs only a mild symptomatic treatment can be expected to occur, while aducanumab has only a marginal, although assumedly causative therapeutic effect. The chronic ingestion of caffeine, a most consumed preferential A2AR antagonist contained in coffee, results in decreased hippocampal tau hyperphosphorylation and neuroinflammation, as well as improvement of memory deficits [45, 249]. Accordingly, selective A2AR antagonists, such as istradefylline, applied over a short period (3 weeks) was found to restore memory performance in different rodent models of amyloid pathology [150, 250]. In consequence, there is sufficient experimental evidence to turn to the US FDA approved drug istradefylline (Nourianz) in clinical studies to investigate its possible anti‐AD effect.

In fact, istradefylline has been introduced as an adjuvant therapy to L‐DOPA in PD [251, 252]. Motor deficits characteristic of PD involve an overactivation of the striatopallidal afferent pathway of the dorsal striatum, due to the degeneration of dopaminergic projection neurons in the substantia nigra pars compacta [246]. A2AR antagonists inhibit striatal D2R binding, possibly through A2AR‐D2R heteromers [253]. While PD is primarily characterized by motor symptoms, cognitive dysfunction also occurs both in the early and later stages of the disease process [254]. Recent studies demonstrated that the A2AR antagonists reversed working memory impairments in animal models of PD [255]. Unfortunately, the effect of istradefylline on cognition was hitherto not tested in PD patients; this however, should be made up leeway in the future.

## 7 Conclusions and Prospects

In the CNS, purinergic cotransmission may modulate the release/effect of the main transmitters (e.g., glutamate, GABA), but the primary modulatory function in this respect appears to be reserved for astrocyte‐derived ATP and its enzymatic degradation product adenosine. Two important functions of the brain, cognition, and emotion are intimately regulated by the gliotransmitters ATP/adenosine. While cognitive and emotional disturbances are typical human disorders, experimental medicine makes use of various animal models, which, although incompletely, are able to model these diseases. In consequence, the aim of the present review was to elucidate the involvement of astrocyte‐neuron interactions via the gliotransmitter ATP and its metabolite adenosine, in hippocampal and prefronto‐cortical cognitive and affective processes.

Repetitive stimulation of the neuronal input of many synapses in the CNS results in LTP/LTD, regarded as cellular equivalent for learning and memory. ATP and adenosine can up‐ or downregulate both the changes in synaptic strength and the consequent learning performance in the respective animal (usually rodent) models. The hippocampus, which acts as a sieve and transformer for stimuli entering from the entorhinal cortex this tri‐neuronal system (dentate gyrus, CA3, and CA1 pyramidal cells) and leaving it via axonal projections to various areas of the brain, is crucially involved in cognitive and emotional regulation. In the case of affective disorders (MDD, bipolar disorder), the eminent significance of a unidirectional projection connecting the hippocampus with the mPFC has to be taken into consideration. Difficulties encountered with the use of rodent models are still more prominent in relation to affective disorders, than in case of the cognitive ones. Most of these latter models are based on the application of acute stressful stimuli, although the human disorder MDD is caused by the interplay of chronic environmental/social stress and genetic factors. We pay particular attention to animal models because the present review reports data raised experimentally and only by extrapolation generates clinically relevant data.

After recognizing astrocytic modulation by the “purinome” (purinergic transmitters, their astrocytic release mechanisms, their receptors and synthesizing/decomposing enzymes) of cognitive processes, we discussed the dual hypothesis of depression‐like behavior by increased or decreased levels of ATP in the hippocampus/mPFC. Both of these opposing changes might be of astrocytic origin, although the former is due to P2X7R‐mediated modulation of pyramidal cell function, while the latter is due to activation of the apoptotic/necrotic/inflammatory P2X7R. The involvement of astrocyte‐derived adenosine in cognitive deterioration or affective disorders has been hitherto proven only in a few cases, but this might be due to the limited interest directed to this specific question.

Animal experimentation has always the aim of clarifying some clinically important issues, and, therefore, the numerous *caveats* associated with the animal models are an apparent drawback. Thus, on the one hand, further clinical studies and work with *postmortem* human material is expected to generate relevant findings, and on the other hand, new techniques on the subcellular (transcriptomics, metabolomics), but also on the whole animal/human level (refined imaging methods) are expected to become particularly helpful. Eventually, a new and most promising field is the measurement of blood plasma levels of sP2X7Rs as a diagnostic tool to identify (neuro)inflammation.

## Author Contributions

Peter Illes has written the first version of the manuscript. Patrizia Rubini, Henning Ulrich, Hai‐Yan Yin, and Yong Tang improved and extended the paper. All authors consented with the final version.

## Ethics Statement

The authors have nothing to report.

## Conflicts of Interest

Author Peter Illes is an Editorial Board Member of MedComm. He was not involved in the journal's review of or decision related to this manuscript. All authors declared no conflicts of interest.

## Data Availability

The authors have nothing to report.

## References

[1] G. Perea , M. Sur , and A. Araque , “Neuron‐Glia Networks: Integral Gear of Brain Function,” Frontiers in Cellular Neuroscience 8 (2014): 378. 25414643 10.3389/fncel.2014.00378 PMC4222327

[2] B. S. Khakh , “Astrocyte‐Neuron Interactions in the Striatum: Insights on Identity, Form, and Function,” Trends in Neuroscience (Tins) 42, no. 9 (2019): 617–630. 10.1016/j.tins.2019.06.003 PMC6741427 31351745

[3] L. K. Bak , A. Schousboe , and H. S. Waagepetersen , “The Glutamate/GABA‐Glutamine Cycle: Aspects of Transport, Neurotransmitter Homeostasis and Ammonia Transfer,” Journal of Neurochemistry 98, no. 3 (2006): 641–653. 16787421 10.1111/j.1471-4159.2006.03913.x

[4] E. A. Nagelhus and O. P. Ottersen , “Physiological Roles of Aquaporin‐4 in Brain,” Physiological Reviews 93, no. 4 (2013): 1543–1562. 24137016 10.1152/physrev.00011.2013 PMC3858210

[5] N. B. Hamilton and D. Attwell , “Do Astrocytes Really Exocytose Neurotransmitters?,” Nature Reviews Neuroscience 11, no. 4 (2010): 227–238. 20300101 10.1038/nrn2803

[6] A. Araque , G. Carmignoto , P. G. Haydon , S. H. R. Oliet , R. Robitaille , and A. Volterra , “Gliotransmitters Travel in Time and Space,” Neuron 81, no. 4 (2014): 728–739. 24559669 10.1016/j.neuron.2014.02.007 PMC4107238

[7] M. M. Halassa , T. Fellin , and P. G. Haydon , “Tripartite Synapses: Roles for Astrocytic Purines in the Control of Synaptic Physiology and Behavior,” Neuropharmacology 57, no. 4 (2009): 343–346. 19577581 10.1016/j.neuropharm.2009.06.031 PMC3190118

[8] A. Mishra , J. P. Reynolds , Y. Chen , A. V. Gourine , D. A. Rusakov , and D. Attwell , “Astrocytes Mediate Neurovascular Signaling to Capillary Pericytes but Not to Arterioles,” Nature Neuroscience 19, no. 12 (2016): 1619–1627. 27775719 10.1038/nn.4428 PMC5131849

[9] E. Scharbarg , A. Walter , L. Lecoin , et al., “Prostaglandin D(2) Controls Local Blood Flow and Sleep‐Promoting Neurons in the VLPO via Astrocyte‐Derived Adenosine,” Acs Chemical Neuroscience 14, no. 6 (2023): 1063–1070. 36847485 10.1021/acschemneuro.2c00660

[10] J. A. Wells , I. N. Christie , P. S. Hosford , et al., “A Critical Role for Purinergic Signalling in the Mechanisms Underlying Generation of BOLD fMRI Responses,” Journal of Neuroscience 35, no. 13 (2015): 5284–5292. 25834053 10.1523/JNEUROSCI.3787-14.2015 PMC4381001

[11] M. M. Halassa , C. Florian , T. Fellin , et al., “Astrocytic Modulation of Sleep Homeostasis and Cognitive Consequences of Sleep Loss,” Neuron 61, no. 2 (2009): 213–219. 19186164 10.1016/j.neuron.2008.11.024 PMC2673052

[12] T. Fujita , M. J. Chen , B. Li , et al., “Neuronal Transgene Expression in Dominant‐Negative SNARE Mice,” Journal of Neuroscience 34, no. 50 (2014): 16594–16604. 25505312 10.1523/JNEUROSCI.2585-14.2014 PMC4261088

[13] X. Cao , L.‐P. Li , Q. Wang , et al., “Astrocyte‐Derived ATP Modulates Depressive‐Like Behaviors,” Nature Medicine 19, no. 6 (2013): 773–777. 10.1038/nm.3162 23644515

[14] Y. Pankratov and U. Lalo , “Role for Astroglial α1‐Adrenoreceptors in Gliotransmission and Control of Synaptic Plasticity in the Neocortex,” Frontiers in Cellular Neuroscience 9 (2015): 230. 26136663 10.3389/fncel.2015.00230 PMC4468378

[15] V. Ralevic and W. R. Dunn , “Purinergic Transmission in Blood Vessels,” Autonomic Neuroscience 191 (2015): 48–66. 26004513 10.1016/j.autneu.2015.04.007

[16] W. Nörenberg and P. Illes , “Neuronal P2X Receptors: Localisation and Functional Properties,” Naunyn‐Schmiedebergs Archives of Pharmacology 362, no. 4‐5 (2000): 324–339. 11111827 10.1007/s002100000311

[17] Z. Zhang , G. Chen , W. Zhou , et al., “Regulated ATP Release From Astrocytes Through Lysosome Exocytosis,” Nature Cell Biology 9, no. 8 (2007): 945–953. 17618272 10.1038/ncb1620

[18] A. Verkhratsky , M. Matteoli , V. Parpura , J.‐P. Mothet , and R. Zorec , “Astrocytes as Secretory Cells of the Central Nervous System: Idiosyncrasies of Vesicular Secretion,” Embo Journal 35, no. 3 (2016): 239–257. 26758544 10.15252/embj.201592705 PMC4741299

[19] M. W. Sherwood , M. Arizono , A. Panatier , K. Mikoshiba , and S. H. R. Oliet , “Astrocytic IP(3)Rs: Beyond IP(3)R2,” Frontiers in Cellular Neuroscience 15 (2021): 695817. 34393726 10.3389/fncel.2021.695817 PMC8363081

[20] E. Pryazhnikov and L. Khiroug , “Sub‐Micromolar Increase in Ca(2+)(i) Triggers Delayed Exocytosis of ATP in Cultured Astrocytes,” Glia 56, no. 1 (2008): 38–49. 17910050 10.1002/glia.20590

[21] T. Liu , L. Sun , Y. Xiong , et al., “Calcium Triggers Exocytosis From Two Types of Organelles in a Single Astrocyte,” Journal of Neuroscience 31, no. 29 (2011): 10593–10601. 21775603 10.1523/JNEUROSCI.6401-10.2011 PMC6622647

[22] M. Kreft , M. Stenovec , M. Rupnik , et al., “Properties of Ca(2+)‐Dependent Exocytosis in Cultured Astrocytes,” Glia 46, no. 4 (2004): 437–445. 15095373 10.1002/glia.20018

[23] M. Oya , T. Kitaguchi , Y. Yanagihara , et al., “Vesicular Nucleotide Transporter Is Involved in ATP Storage of Secretory Lysosomes in Astrocytes,” Biochemical and Biophysical Research Communications 438, no. 1 (2013): 145–151. 23876310 10.1016/j.bbrc.2013.07.043

[24] L. Xing , T. Yang , S. Cui , and G. Chen , “Connexin Hemichannels in Astrocytes: Role in CNS Disorders,” Frontiers in Molecular Neuroscience 12 (2019): 23. 30787868 10.3389/fnmol.2019.00023 PMC6372977

[25] S. O. Suadicani , R. Iglesias , J. Wang , G. Dahl , D. C. Spray , and E. Scemes , “ATP Signaling Is Deficient in Cultured Pannexin1‐Null Mouse Astrocytes,” Glia 60, no. 7 (2012): 1106–1116. 22499153 10.1002/glia.22338 PMC3348971

[26] R. Z. Sabirov , M. R. Islam , T. Okada , et al., “The ATP‐Releasing Maxi‐Cl Channel: Its Identity, Molecular Partners and Physiological/Pathophysiological Implications,” Life (Basel) 11, no. 6 (2021): 509. 34073084 10.3390/life11060509 PMC8229958

[27] H.‐T. Liu , R. Z. Sabirov , and Y. Okada , “Oxygen‐Glucose Deprivation Induces ATP Release via Maxi‐Anion Channels in Astrocytes,” Purinergic Signalling 4, no. 2 (2008): 147–154. 18368522 10.1007/s11302-007-9077-8 PMC2377326

[28] S. Lee , B.‐E. Yoon , K. Berglund , et al., “Channel‐Mediated Tonic GABA Release From Glia,” Science 330, no. 6005 (2010): 790–796. 20929730 10.1126/science.1184334

[29] G. Lazutkaite , A. Soldà , K. Lossow , W. Meyerhof , and N. Dale , “Amino Acid Sensing in Hypothalamic Tanycytes via Umami Taste Receptors,” Molecular Metabolism 6, no. 11 (2017): 1480–1492. 29107294 10.1016/j.molmet.2017.08.015 PMC5681271

[30] S. Duan , C. M. Anderson , E. C. Keung , Y. Chen , Y. Chen , and R. A. Swanson , “P2×7 Receptor‐Mediated Release of Excitatory Amino Acids From Astrocytes,” Journal of Neuroscience 23, no. 4 (2003): 1320–1328. 12598620 10.1523/JNEUROSCI.23-04-01320.2003 PMC6742264

[31] P. Illes , T. M. Khan , and P. Rubini , “Neuronal P2×7 Receptors Revisited: Do They Really Exist?,” Journal of Neuroscience 37, no. 30 (2017): 7049–7062. 28747388 10.1523/JNEUROSCI.3103-16.2017 PMC6705732

[32] J. Lezmy , “How Astrocytic ATP Shapes Neuronal Activity and Brain Circuits,” Current Opinion in Neurobiology 79 (2023): 102685. 36746109 10.1016/j.conb.2023.102685

[33] U. Lalo and Y. Pankratov , “ATP‐Mediated Signalling in the Central Synapses,” Neuropharmacology 229 (2023): 109477. 36841527 10.1016/j.neuropharm.2023.109477

[34] H. Zimmermann , M. Zebisch , and N. Sträter , “Cellular Function and Molecular Structure of Ecto‐Nucleotidases,” Purinergic Signalling 8, no. 3 (2012): 437–502. 22555564 10.1007/s11302-012-9309-4 PMC3360096

[35] G. G. Yegutkin , “Enzymes Involved in Metabolism of Extracellular Nucleotides and Nucleosides: Functional Implications and Measurement of Activities,” Critical Reviews in Biochemistry and Molecular Biology 49, no. 6 (2014): 473–497. 25418535 10.3109/10409238.2014.953627

[36] M. Wall and N. Dale , “Activity‐Dependent Release of Adenosine: A Critical Re‐Evaluation of Mechanism,” Current Neuropharmacology 6, no. 4 (2008): 329–337. 19587854 10.2174/157015908787386087 PMC2701281

[37] A. Bicket , P. Mehrabi , Z. Naydenova , et al., “Novel Regulation of Equlibrative Nucleoside Transporter 1 (ENT1) by Receptor‐Stimulated Ca2+‐Dependent Calmodulin Binding,” American Journal of Physiology. Cell Physiology 310, no. 10 (2016): C808–820. 27009875 10.1152/ajpcell.00243.2015 PMC4895448

[38] M. P. Abbracchio and G. Burnstock , “Purinoceptors: Are There Families of P2X and P2Y Purinoceptors?,” Pharmacology & Therapeutics 64, no. 3 (1994): 445–475. 7724657 10.1016/0163-7258(94)00048-4

[39] M. P. Abbracchio , G. Burnstock , A. Verkhratsky , and H. Zimmermann , “Purinergic Signalling in the Nervous System: An Overview,” Trends in Neuroscience (Tins) 32, no. 1 (2009): 19–29. 10.1016/j.tins.2008.10.001 19008000

[40] A. Rivera , I. Vanzulli , and A. M. Butt , “A Central Role for ATP Signalling in Glial Interactions in the CNS,” Current Drug Targets 17, no. 16 (2016): 1829–1833. 27400972 10.2174/1389450117666160711154529

[41] A. M. Butt , “ATP: A Ubiquitous Gliotransmitter Integrating Neuron‐Glial Networks,” Seminars in Cell & Developmental Biology 22, no. 2 (2011): 205–213. 21376829 10.1016/j.semcdb.2011.02.023

[42] P. Illes , C. E. Müller , K. A. Jacobson , et al., “Update of P2X Receptor Properties and Their Pharmacology: IUPHAR Review 30,” British Journal of Pharmacology 178, no. 3 (2021): 489–514. 33125712 10.1111/bph.15299 PMC8199792

[43] Z. Huang , N. Xie , P. Illes , et al., “From Purines to Purinergic Signalling: Molecular Functions and Human Diseases,” Signal Transduction and Targeted Therapy 6, no. 1 (2021): 162. 33907179 10.1038/s41392-021-00553-z PMC8079716

[44] M. P. Abbracchio , G. Burnstock , J.‐M. Boeynaems , et al., “International Union of Pharmacology LVIII: Update on the P2Y G Protein‐Coupled Nucleotide Receptors: From Molecular Mechanisms and Pathophysiology to Therapy,” Pharmacological Reviews 58, no. 3 (2006): 281–341. 16968944 10.1124/pr.58.3.3 PMC3471216

[45] K. A. Jacobson , E. G. Delicado , C. Gachet , et al., “Update of P2Y Receptor Pharmacology: IUPHAR Review 27,” British Journal of Pharmacology 177, no. 11 (2020): 2413–2433. 32037507 10.1111/bph.15005 PMC7205808

[46] B. B. Fredholm , A. P. IJzerman , K. A. Jacobson , J. Linden , and C. E. Müller , “International Union of Basic and Clinical Pharmacology. LXXXI. Nomenclature and Classification of Adenosine Receptors–An Update,” Pharmacological Reviews 63, no. 1 (2011): 1–34. 21303899 10.1124/pr.110.003285 PMC3061413

[47] A. P. IJzerman , K. A. Jacobson , C. E. Müller , B. N. Cronstein , and R. A. Cunha , “International Union of Basic and Clinical Pharmacology. CXII: Adenosine Receptors: A Further Update,” Pharmacological Reviews 74, no. 2 (2022): 340–372. 35302044 10.1124/pharmrev.121.000445 PMC8973513

[48] J.‐F. Chen , “Adenosine Receptor Control of Cognition in Normal and Disease,” International Review of Neurobiology 119 (2014): 257–307. 25175970 10.1016/B978-0-12-801022-8.00012-X

[49] C. P. Garcia , A. Licht‐Murava , and A. G. Orr , “Effects of Adenosine A(2A) Receptors on Cognitive Function in Health and Disease,” International Review of Neurobiology 170 (2023): 121–154. 37741689 10.1016/bs.irn.2023.04.006

[50] S. M. Naes , S. Ab‐Rahim , M. Mazlan , and A. Abdul Rahman , “Equilibrative Nucleoside Transporter 2: Properties and Physiological Roles,” BioMed Research International 2020 (2020): 5197626. 33344638 10.1155/2020/5197626 PMC7732376

[51] D. Boison and M. F. Jarvis , “Adenosine Kinase: A Key Regulator of Purinergic Physiology,” Biochemical Pharmacology 187 (2021): 114321. 33161022 10.1016/j.bcp.2020.114321 PMC8096637

[52] D. Boison and E. Aronica , “Comorbidities in Neurology: Is Adenosine the Common Link?,” Neuropharmacology 97 (2015): 18–34. 25979489 10.1016/j.neuropharm.2015.04.031 PMC4537378

[53] B. Sperlágh , E. S. Vizi , K. Wirkner , and P. Illes , “P2×7 Receptors in the Nervous System,” Progress in Neurobiology 78, no. 6 (2006): 327–346. 16697102 10.1016/j.pneurobio.2006.03.007

[54] Y. Zhang , H.‐Y. Yin , P. Rubini , Y. Tang , and P. Illes , “A Possible Causal Involvement of Neuroinflammatory, Purinergic P2×7 Receptors in Psychiatric Disorders,” Current Neuropharmacology 20, no. 11 (2022): 2142–2155. 35236262 10.2174/1570159X20666220302152400 PMC9886837

[55] A. Surprenant , F. Rassendren , E. Kawashima , R. A. North , and G. Buell , “The Cytolytic P2Z Receptor for Extracellular ATP Identified as a P2X Receptor (P2×7),” Science 272, no. 5262 (1996): 735–738. 8614837 10.1126/science.272.5262.735

[56] F. Di Virgilio , D. Dal Ben , A. C. Sarti , A. L. Giuliani , and S. Falzoni , “The P2×7 Receptor in Infection and Inflammation,” Immunity 47, no. 1 (2017): 15–31. 28723547 10.1016/j.immuni.2017.06.020

[57] R. Sluyter , “The P2×7 Receptor,” Advances in Experimental Medicine and Biology 1051 (2017): 17–53. 28676924 10.1007/5584_2017_59

[58] P. Illes , “P2×7 Receptors Amplify CNS Damage in Neurodegenerative Diseases,” International Journal of Molecular Sciences 21, no. 17 (2020): 5996. 32825423 10.3390/ijms21175996 PMC7504621

[59] J. L. Voss , D. J. Bridge , N. J. Cohen , and J. A. Walker , “A Closer Look at the Hippocampus and Memory,” Trends in Cognitive Sciences 21, no. 8 (2017): 577–588. 28625353 10.1016/j.tics.2017.05.008 PMC5659202

[60] J. Lisman , G. Buzsáki , H. Eichenbaum , L. Nadel , C. Ranganath , and A. D. Redish , “Viewpoints: How the Hippocampus Contributes to Memory, Navigation and Cognition,” Nature Neuroscience 20, no. 11 (2017): 1434–1447. 29073641 10.1038/nn.4661 PMC5943637

[61] C. A. Stockmeier , G. J. Mahajan , L. C. Konick , et al., “Cellular Changes in the Postmortem Hippocampus in Major Depression,” Biological Psychiatry 56, no. 9 (2004): 640–650. 15522247 10.1016/j.biopsych.2004.08.022 PMC2929806

[62] G. MacQueen and T. Frodl , “The Hippocampus in Major Depression: Evidence for the Convergence of the Bench and Bedside in Psychiatric Research?,” Molecular Psychiatry 16, no. 3 (2011): 252–264. 20661246 10.1038/mp.2010.80

[63] H. Hagena and D. Manahan‐Vaughan , “Interplay of Hippocampal Long‐Term Potentiation and Long‐Term Depression in Enabling Memory Representations,” Philosophical Transactions of the Royal Society of London. Series B: Biological Sciences 379, no. 1906 (2024): 20230229. 38853558 10.1098/rstb.2023.0229 PMC11343234

[64] M. A. Rogers , K. Kasai , M. Koji , et al., “Executive and Prefrontal Dysfunction in Unipolar Depression: A Review of Neuropsychological and Imaging Evidence,” Neuroscience Research 50, no. 1 (2004): 1–11. 15288493 10.1016/j.neures.2004.05.003

[65] H. J. Kang , B. Voleti , T. Hajszan , et al., “Decreased Expression of Synapse‐Related Genes and Loss of Synapses in Major Depressive Disorder,” Nature Medicine 18, no. 9 (2012): 1413–1417. 10.1038/nm.2886 PMC3491115 22885997

[66] B. P. Godsil , J. P. Kiss , M. Spedding , and T. M. Jay , “The Hippocampal‐Prefrontal Pathway: The Weak Link in Psychiatric Disorders?,” European Neuropsychopharmacology 23, no. 10 (2013): 1165–1181. 23332457 10.1016/j.euroneuro.2012.10.018

[67] Y. H. Ahn , Y. Tang , and P. Illes , “The Neuroinflammatory Astrocytic P2×7 Receptor: Alzheimer's Disease, Ischemic Brain Injury, and Epileptic State,” Expert Opinion on Therapeutic Targets 27, no. 9 (2023): 763–778. 37712394 10.1080/14728222.2023.2258281

[68] Y. Shen , S. M. Specht , I. de Saint Ghislain , and R. Li , “The Hippocampus: A Biological Model for Studying Learning and Memory,” Progress in Neurobiology 44, no. 5 (1994): 485–496. 7886236 10.1016/0301-0082(94)90008-6

[69] L. R. Squire and S. Zola‐Morgan , “The Medial Temporal Lobe Memory System,” Science 253, no. 5026 (1991): 1380–1386. 1896849 10.1126/science.1896849

[70] N. Cowan , “What Are the Differences Between Long‐Term, Short‐Term, and Working Memory?,” Progress in Brain Research 169 (2008): 323–338. 18394484 10.1016/S0079-6123(07)00020-9 PMC2657600

[71] A. Kol , A. Adamsky , M. Groysman , T. Kreisel , M. London , and I. Goshen , “Astrocytes Contribute to Remote Memory Formation by Modulating Hippocampal‐Cortical Communication During Learning,” Nature Neuroscience 23, no. 10 (2020): 1229–1239. 32747787 10.1038/s41593-020-0679-6 PMC7611962

[72] R. Martín , R. Bajo‐Grañeras , R. Moratalla , G. Perea , and A. Araque , “Circuit‐Specific Signaling in Astrocyte‐Neuron Networks in Basal Ganglia Pathways,” Science 349, no. 6249 (2015): 730–734. 26273054 10.1126/science.aaa7945

[73] M. Martin‐Fernandez , S. Jamison , L. M. Robin , et al., “Synapse‐Specific Astrocyte Gating of Amygdala‐Related Behavior,” Nature Neuroscience 20, no. 11 (2017): 1540–1548. 28945222 10.1038/nn.4649 PMC5903286

[74] A. Adamsky , A. Kol , T. Kreisel , et al., “Astrocytic Activation Generates De Novo Neuronal Potentiation and Memory Enhancement,” Cell 174, no. 1 (2018): 59–71.e14. 29804835 10.1016/j.cell.2018.05.002

[75] R. M. Church , “Quantitative Models of Animal Learning and Cognition,” Journal of Experimental Psychology: Animal Behavior Processes 23, no. 4 (1997): 379–389. 9335132 10.1037//0097-7403.23.4.379

[76] Neha , R. K. Sodhi , A. S. Jaggi , and N. Singh , “Animal Models of Dementia and Cognitive Dysfunction,” Life Sciences 109, no. 2 (2014): 73–86. 25066372 10.1016/j.lfs.2014.05.017

[77] P. Illes , H. Ulrich , J.‐F. Chen , and Y. Tang , “Purinergic Receptors in Cognitive Disturbances,” Neurobiology of Disease 185 (2023): 106229. 37453562 10.1016/j.nbd.2023.106229

[78] E. Decrock , M. de Bock , N. Wang , et al., “Connexin and Pannexin Signaling Pathways, an Architectural Blueprint for CNS Physiology and Pathology?,” Cellular and Molecular Life Sciences 72, no. 15 (2015): 2823–2851. 26118660 10.1007/s00018-015-1962-7 PMC11113968

[79] P. Illes , P. Rubini , L. Huang , and Y. Tang , “The P2×7 Receptor: A New Therapeutic Target in Alzheimer's Disease,” Expert Opinion on Therapeutic Targets 23, no. 3 (2019): 165–176. 30691318 10.1080/14728222.2019.1575811

[80] C. J. Gallagher and M. W. Salter , “Differential Properties of Astrocyte Calcium Waves Mediated by P2Y1 and P2Y2 Receptors,” Journal of Neuroscience 23, no. 17 (2003): 6728–6739. 12890765 10.1523/JNEUROSCI.23-17-06728.2003 PMC6740738

[81] J.‐T. He , X.‐Y. Li , L. Yang , and X. Zhao , “Astroglial Connexins and Cognition: Memory Formation or Deterioration?,” Bioscience Reports 40, no. 1 (2020): BSR20193510. 31868207 10.1042/BSR20193510 PMC6954363

[82] O. D. Jones , S. R. Hulme , and W. C. Abraham , “Purinergic Receptor‐ and Gap Junction‐Mediated Intercellular Signalling as a Mechanism of Heterosynaptic Metaplasticity,” Neurobiology of Learning and Memory 105 (2013): 31–39. 23747410 10.1016/j.nlm.2013.05.010

[83] L. Walrave , M. Vinken , G. Albertini , D. de Bundel , L. Leybaert , and I. J. Smolders , “Inhibition of Connexin43 Hemichannels Impairs Spatial Short‐Term Memory Without Affecting Spatial Working Memory,” Frontiers in Cellular Neuroscience 10 (2016): 288. 28066184 10.3389/fncel.2016.00288 PMC5168429

[84] W. Zhang , J. Yin , B.‐Y. Gao , et al., “Inhibition of Astroglial Hemichannels Ameliorates Infrasonic Noise Induced Short‐Term Learning and Memory Impairment,” Behavioral and Brain Functions 19, no. 1 (2023): 23. 38110991 10.1186/s12993-023-00226-7 PMC10726613

[85] N. Prochnow , A. Abdulazim , S. Kurtenbach , et al., “Pannexin1 Stabilizes Synaptic Plasticity and Is Needed for Learning,” PLoS ONE 7, no. 12 (2012): e51767. 23284764 10.1371/journal.pone.0051767 PMC3527502

[86] P. Obot , G. Subah , A. Schonwald , et al., “Astrocyte and Neuronal Panx1 Support Long‐Term Reference Memory in Mice,” ASN Neuro 15 (2023): 17590914231184712. 37365910 10.1177/17590914231184712 PMC10326369

[87] P. Pelegrin and A. Surprenant , “Pannexin‐1 Mediates Large Pore Formation and Interleukin‐1beta Release by the ATP‐Gated P2×7 Receptor,” Embo Journal 25, no. 21 (2006): 5071–5082. 17036048 10.1038/sj.emboj.7601378 PMC1630421

[88] P. Pelegrin , “Targeting Interleukin‐1 Signaling in Chronic Inflammation: Focus on P2X(7) Receptor and Pannexin‐1,” Drug News & Perspectives 21, no. 8 (2008): 424–433. 19034348 10.1358/dnp.2008.21.8.1265800

[89] M. T. Khan , J. Deussing , Y. Tang , and P. Illes , “Astrocytic Rather Than Neuronal P2×7 Receptors Modulate the Function of the Tri‐Synaptic Network in the Rodent Hippocampus,” Brain Research Bulletin 151 (2019): 164–173. 30098388 10.1016/j.brainresbull.2018.07.016

[90] K. Kaczmarek‐Hajek , J. Zhang , R. Kopp , et al., “Re‐Evaluation of Neuronal P2×7 Expression Using Novel Mouse Models and a P2×7‐Specific Nanobody,” Elife 7 (2018): e36217. 30074479 10.7554/eLife.36217 PMC6140716

[91] A. Ramírez‐Fernández , L. Urbina‐Treviño , G. Conte , et al., “Deviant Reporter Expression and P2×4 Passenger Gene Overexpression in the Soluble EGFP BAC Transgenic P2×7 Reporter Mouse Model,” Scientific Reports 10, no. 1 (2020): 19876. 33199725 10.1038/s41598-020-76428-0 PMC7669894

[92] S. G. Anagnostaras , G. D. Gale , and M. S. Fanselow , “Hippocampus and Contextual Fear Conditioning: Recent Controversies and Advances,” Hippocampus 11, no. 1 (2001): 8–17. 11261775 10.1002/1098-1063(2001)11:1<8::AID-HIPO1015>3.0.CO;2-7

[93] N. G. Fiorenza , J. Rosa , I. Izquierdo , and J. C. Myskiw , “Modulation of the Extinction of Two Different Fear‐Motivated Tasks in Three Distinct Brain Areas,” Behavioural Brain Research 232, no. 1 (2012): 210–216. 22525015 10.1016/j.bbr.2012.04.015

[94] L. B. Domingos , S. C. Hott , A. L. B. Terzian , and L. B. M. Resstel , “P2×7 Purinergic Receptors Participate in the Expression and Extinction Processes of Contextual Fear Conditioning Memory in Mice,” Neuropharmacology 128 (2018): 474–481. 28802645 10.1016/j.neuropharm.2017.08.005

[95] R. C. Campos , G. M. Parfitt , C. E. Polese , R. Coutinho‐Silva , F. B. Morrone , and D. M. Barros , “Pharmacological Blockage and P2×7 Deletion Hinder Aversive Memories: Reversion in an Enriched Environment,” Neuroscience 280 (2014): 220–230. 25239372 10.1016/j.neuroscience.2014.09.017

[96] P. Illes , G. Burnstock , and Y. Tang , “Astroglia‐Derived ATP Modulates CNS Neuronal Circuits,” Trends in Neuroscience (Tins) 42, no. 12 (2019): 885–898. 10.1016/j.tins.2019.09.006 31704181

[97] K. Farrell , M. Musaus , S. Navabpour , et al., “Proteomic Analysis Reveals Sex‐Specific Protein Degradation Targets in the Amygdala During Fear Memory Formation,” Frontiers in Molecular Neuroscience 14 (2021): 716284. 34658783 10.3389/fnmol.2021.716284 PMC8511838

[98] S. Bissiere , M. Zelikowsky , R. Ponnusamy , N. S. Jacobs , H. T. Blair , and Fanselow , “Electrical Synapses Control Hippocampal Contributions to Fear Learning and Memory,” Science 331, no. 6013 (2011): 87–91. 21212357 10.1126/science.1193785 PMC4276370

[99] J. Stehberg , R. Moraga‐Amaro , C. Salazar , et al., “Release of Gliotransmitters Through Astroglial Connexin 43 Hemichannels Is Necessary for Fear Memory Consolidation in the Basolateral Amygdala,” Faseb Journal 26, no. 9 (2012): 3649–3657. 22665389 10.1096/fj.11-198416

[100] J. Götz , L.‐G. Bodea , and M. Goedert , “Rodent Models for Alzheimer Disease,” Nature Reviews Neuroscience 19, no. 10 (2018): 583–598. 30194347 10.1038/s41583-018-0054-8

[101] L. C. Dos Santos Picanco , P. F. Ozela , M. de Fatima de Brito Brito , et al., “Alzheimer's Disease: A Review From the Pathophysiology to Diagnosis, New Perspectives for Pharmacological Treatment,” Current Medicinal Chemistry 25, no. 26 (2018): 3141–3159. 30191777 10.2174/0929867323666161213101126

[102] D. E. Ribeiro , A. L. Roncalho , T. Glaser , H. Ulrich , G. Wegener , and S. Joca , “P2×7 Receptor Signaling in Stress and Depression,” International Journal of Molecular Sciences 20, no. 11 (2019): 2778. 31174279 10.3390/ijms20112778 PMC6600521

[103] Q. Huang , J. Ying , W. Yu , et al., “P2×7 Receptor: An Emerging Target in Alzheimer's Disease,” Molecular Neurobiology 61, no. 5 (2024): 2866–2880. 37940779 10.1007/s12035-023-03699-9 PMC11043177

[104] E. Martin , M. Amar , C. Dalle , et al., “New Role of P2×7 Receptor in an Alzheimer's Disease Mouse Model,” Molecular Psychiatry 24, no. 1 (2019): 108–125. 29934546 10.1038/s41380-018-0108-3 PMC6756107

[105] A. Delekate , M. Füchtemeier , T. Schumacher , C. Ulbrich , M. Foddis , and G. C. Petzold , “Metabotropic P2Y1 Receptor Signalling Mediates Astrocytic Hyperactivity in Vivo in an Alzheimer's Disease Mouse Model,” Nature Communications 5 (2014): 5422. 10.1038/ncomms6422 25406732

[106] N. Reichenbach , A. Delekate , B. Breithausen , et al., “P2Y1 Receptor Blockade Normalizes Network Dysfunction and Cognition in an Alzheimer's Disease Model,” Journal of Experimental Medicine 215, no. 6 (2018): 1649–1663. 29724785 10.1084/jem.20171487 PMC5987918

[107] X. Mei , P. Ezan , C. Giaume , and A. Koulakoff , “Astroglial Connexin Immunoreactivity Is Specifically Altered at β‐Amyloid Plaques in β‐Amyloid Precursor Protein/presenilin1 Mice,” Neuroscience 171, no. 1 (2010): 92–105. 20813165 10.1016/j.neuroscience.2010.08.001

[108] R. Ren , L. Zhang , and M. Wang , “Specific Deletion Connexin43 in Astrocyte Ameliorates Cognitive Dysfunction in APP/PS1 Mice,” Life Sciences 208 (2018): 175–191. 30031059 10.1016/j.lfs.2018.07.033

[109] J. I. Nagy , W. Li , E. L. Hertzberg , and C. A. Marotta , “Elevated Connexin43 Immunoreactivity at Sites of Amyloid Plaques in Alzheimer's Disease,” Brain Research 717, no. 1‐2 (1996): 173–178. 8738268 10.1016/0006-8993(95)01526-4

[110] Y. Pankratov , U. Lalo , O. A. Krishtal , and A. Verkhratsky , “P2X Receptors and Synaptic Plasticity,” Neuroscience 158, no. 1 (2009): 137–148. 18495357 10.1016/j.neuroscience.2008.03.076

[111] S. J. Guzman and Z. Gerevich , “P2Y Receptors in Synaptic Transmission and Plasticity: Therapeutic Potential in Cognitive Dysfunction,” Neural Plasticity 2016 (2016): 1207393. 27069691 10.1155/2016/1207393 PMC4812485

[112] E. Boué‐Grabot and Y. Pankratov , “Modulation of Central Synapses by Astrocyte‐Released ATP and Postsynaptic P2X Receptors,” Neural Plasticity 2017 (2017): 9454275. 28845311 10.1155/2017/9454275 PMC5563405

[113] Y. V. Pankratov , U. V. Lalo , and O. A. Krishtal , “Role for P2X Receptors in Long‐Term Potentiation,” Journal of Neuroscience 22, no. 19 (2002): 8363–8369. 12351710 10.1523/JNEUROSCI.22-19-08363.2002 PMC6757784

[114] U. Lalo , O. Palygin , A. Verkhratsky , S. G. N. Grant , and Y. Pankratov , “ATP From Synaptic Terminals and Astrocytes Regulates NMDA Receptors and Synaptic Plasticity Through PSD‐95 Multi‐Protein Complex,” Scientific Reports 6 (2016): 33609. 27640997 10.1038/srep33609 PMC5027525

[115] T. Sumi and K. Harada , “Mechanism Underlying Hippocampal Long‐Term Potentiation and Depression Based on Competition Between Endocytosis and Exocytosis of AMPA Receptors,” Scientific Reports 10, no. 1 (2020): 14711. 32895399 10.1038/s41598-020-71528-3 PMC7477194

[116] A. Volianskis , G. France , M. S. Jensen , Z. A. Bortolotto , D. E. Jane , and G. L. Collingridge , “Long‐Term Potentiation and the Role of N‐Methyl‐D‐Aspartate Receptors,” Brain Research 1621 (2015): 5–16. 25619552 10.1016/j.brainres.2015.01.016 PMC4563944

[117] M. Temido‐Ferreira , D. G. Ferreira , V. L. Batalha , et al., “Age‐Related Shift in LTD Is Dependent on Neuronal Adenosine A(2A) Receptors Interplay With mGluR5 and NMDA Receptors,” Molecular Psychiatry 25, no. 8 (2020): 1876–1900. 29950682 10.1038/s41380-018-0110-9 PMC7387321

[118] F. M. Mouro , D. M. Rombo , R. B. Dias , J. A. Ribeiro , and A. M. Sebastião , “Adenosine A(2A) Receptors Facilitate Synaptic NMDA Currents in CA1 Pyramidal Neurons,” British Journal of Pharmacology 175, no. 23 (2018): 4386–4397. 30220081 10.1111/bph.14497 PMC6240125

[119] M. F. Pereira , I. M. Amaral , C. Lopes , et al., “l‐α‐Aminoadipate Causes Astrocyte Pathology With Negative Impact on Mouse Hippocampal Synaptic Plasticity and Memory,” Faseb Journal 35, no. 8 (2021): e21726. 34196433 10.1096/fj.202100336R

[120] U. Lalo and Y. Pankratov , “Astrocyte Ryanodine Receptors Facilitate Gliotransmission and Astroglial Modulation of Synaptic Plasticity,” Frontiers in Cellular Neuroscience 18 (2024): 1382010. 38812795 10.3389/fncel.2024.1382010 PMC11135129

[121] J. Chen , Z. Tan , L. Zeng , et al., “Heterosynaptic Long‐Term Depression Mediated by ATP Released From Astrocytes,” Glia 61, no. 2 (2013): 178–191. 23044720 10.1002/glia.22425

[122] L. Rodriguez , C. Yi , C. Chu , et al., “Cross‐Talk Between P2X and NMDA Receptors,” International Journal of Molecular Sciences 21, no. 19 (2020): 7187. 33003406 10.3390/ijms21197187 PMC7582700

[123] S. Kang , S.‐I. Hong , J. Lee , et al., “Activation of Astrocytes in the Dorsomedial Striatum Facilitates Transition From Habitual to Goal‐Directed Reward‐Seeking Behavior,” Biological Psychiatry 88, no. 10 (2020): 797–808. 32564901 10.1016/j.biopsych.2020.04.023 PMC7584758

[124] A. Pinto‐Duarte , J. E. Coelho , R. A. Cunha , J. A. Ribeiro , and A. M. Sebastião , “Adenosine A2A Receptors Control the Extracellular Levels of Adenosine Through Modulation of Nucleoside Transporters Activity in the Rat Hippocampus,” Journal of Neurochemistry 93, no. 3 (2005): 595–604. 15836618 10.1111/j.1471-4159.2005.03071.x

[125] Z. Wu , Y. Cui , H. Wang , et al., “Neuronal Activity‐Induced, Equilibrative Nucleoside Transporter‐Dependent, Somatodendritic Adenosine Release Revealed by a GRAB Sensor,” Proceeding of the National Academy of Sciences of the United States of America 120, no. 14 (2023): e2212387120. 10.1073/pnas.2212387120 PMC10083574 36996110

[126] X. Sun , L. Dias , C. Peng , et al., “40 Hz Light Flickering Facilitates the Glymphatic Flow via Adenosine Signaling in Mice,” Cell Discovery 10, no. 1 (2024): 81. 39103336 10.1038/s41421-024-00701-z PMC11300858

[127] X. Zhou , Y. He , T. Xu , et al., “40 Hz Light Flickering Promotes Sleep Through Cortical Adenosine Signaling,” Cell Research 34, no. 3 (2024): 214–231. 38332199 10.1038/s41422-023-00920-1 PMC10907382

[128] C.‐C. Lee , C.‐P. Chang , C.‐J. Lin , et al., “Adenosine Augmentation Evoked by an ENT1 Inhibitor Improves Memory Impairment and Neuronal Plasticity in the APP/PS1 Mouse Model of Alzheimer's Disease,” Molecular Neurobiology 55, no. 12 (2018): 8936–8952. 29616397 10.1007/s12035-018-1030-z

[129] C.‐P. Chang , Y.‐G. Chang , P.‐Y. Chuang , et al., “Equilibrative Nucleoside Transporter 1 Inhibition Rescues Energy Dysfunction and Pathology in a Model of Tauopathy,” Acta Neuropathologica Communications 9, no. 1 (2021): 112. 34158119 10.1186/s40478-021-01213-7 PMC8220833

[130] K.‐C. Wu , C.‐Y. Lee , Y. Chern , and C.‐J. Lin , “Amelioration of Lipopolysaccharide‐Induced Memory Impairment in Equilibrative Nucleoside Transporter‐2 Knockout Mice Is Accompanied by the Changes in Glutamatergic Pathways,” Brain, Behavior, and Immunity 96 (2021): 187–199. 34058310 10.1016/j.bbi.2021.05.027

[131] L. Alanko , T. Porkka‐Heiskanen , and S. Soinila , “Localization of Equilibrative Nucleoside Transporters in the Rat Brain,” Journal of Chemical Neuroanatomy 31, no. 3 (2006): 162–168. 16448802 10.1016/j.jchemneu.2005.12.001

[132] F. Q. Gonçalves , J. P. Lopes , H. B. Silva , et al., “Synaptic and Memory Dysfunction in a β‐Amyloid Model of Early Alzheimer's Disease Depends on Increased Formation of ATP‐Derived Extracellular Adenosine,” Neurobiology of Disease 132 (2019): 104570. 31394204 10.1016/j.nbd.2019.104570

[133] A. P. Simões , F. Q. Gonçalves , D. Rial , et al., “CD73‐Mediated Formation of Extracellular Adenosine Is Responsible for Adenosine A(2A) Receptor‐Mediated Control of Fear Memory and Amygdala Plasticity,” International Journal of Molecular Sciences 23, no. 21 (2022): 12826. 36361618 10.3390/ijms232112826 PMC9653840

[134] F. Q. Gonçalves , F. C. Matheus , H. B. Silva , et al., “Increased ATP Release and Higher Impact of Adenosine A(2A) Receptors on Corticostriatal Plasticity in a Rat Model of Presymptomatic Parkinson's Disease,” Molecular Neurobiology 60, no. 3 (2023): 1659–1674. 36547848 10.1007/s12035-022-03162-1 PMC9899190

[135] R. Dang , A. Liu , Y. Zhou , et al., “Astrocytic Neuroligin 3 Regulates Social Memory and Synaptic Plasticity Through Adenosine Signaling in Male Mice,” Nature Communications 15, no. 1 (2024): 8639. 10.1038/s41467-024-52974-3 PMC11452673 39366972

[136] N. Rebola , R. Lujan , R. A. Cunha , and C. Mulle , “Adenosine A2A Receptors Are Essential for Long‐Term Potentiation of NMDA‐EPSCs at Hippocampal Mossy Fiber Synapses,” Neuron 57, no. 1 (2008): 121–134. 18184569 10.1016/j.neuron.2007.11.023

[137] C. Florian , C. G. Vecsey , M. M. Halassa , P. G. Haydon , and T. Abel , “Astrocyte‐Derived Adenosine and A1 Receptor Activity Contribute to Sleep Loss‐Induced Deficits in Hippocampal Synaptic Plasticity and Memory in Mice,” Journal of Neuroscience 31, no. 19 (2011): 6956–6962. 21562257 10.1523/JNEUROSCI.5761-10.2011 PMC3140051

[138] Y. Li , L. Li , J. Wu , et al., “Activation of Astrocytes in Hippocampus Decreases Fear Memory Through Adenosine A(1) Receptors,” Elife 9 (2020): e57155. 32869747 10.7554/eLife.57155 PMC7505657

[139] A. Cavaccini , C. Durkee , P. Kofuji , R. Tonini , and A. Araque , “Astrocyte Signaling Gates Long‐Term Depression at Corticostriatal Synapses of the Direct Pathway,” Journal of Neuroscience 40, no. 30 (2020): 5757–5768. 32541069 10.1523/JNEUROSCI.2369-19.2020 PMC7380972

[140] M. Rivera‐Oliver and M. Díaz‐Ríos , “Using Caffeine and Other Adenosine Receptor Antagonists and Agonists as Therapeutic Tools Against Neurodegenerative Diseases: A Review,” Life Sciences 101, no. 1‐2 (2014): 1–9. 24530739 10.1016/j.lfs.2014.01.083 PMC4115368

[141] J. P. Lopes , A. Pliássova , and R. A. Cunha , “The Physiological Effects of Caffeine on Synaptic Transmission and Plasticity in the Mouse Hippocampus Selectively Depend on Adenosine A(1) and A(2A) Receptors,” Biochemical Pharmacology 166 (2019): 313–321. 31199895 10.1016/j.bcp.2019.06.008

[142] A. Launay , O. Nebie , J. Vijaya Shankara , et al., “The Role of Adenosine A(2A) Receptors in Alzheimer's Disease and Tauopathies,” Neuropharmacology 226 (2023): 109379. 36572177 10.1016/j.neuropharm.2022.109379

[143] D. Madeira , C. R. Lopes , A. P. Simões , P. M. Canas , R. A. Cunha , and P. Agostinho , “Astrocytic A(2A) Receptors Silencing Negatively Impacts Hippocampal Synaptic Plasticity and Memory of Adult Mice,” Glia 71, no. 9 (2023): 2137–2153. 37183905 10.1002/glia.24384

[144] D. Madeira , J. Domingues , C. R. Lopes , P. M. Canas , R. A. Cunha , and P. Agostinho , “Modification of Astrocytic Cx43 Hemichannel Activity in Animal Models of AD: Modulation by Adenosine A(2A) Receptors,” Cellular and Molecular Life Sciences 80, no. 11 (2023): 340. 37898985 10.1007/s00018-023-04983-6 PMC10613596

[145] M. Matos , H.‐Y. Shen , E. Augusto , et al., “Deletion of Adenosine A2A Receptors From Astrocytes Disrupts Glutamate Homeostasis Leading to Psychomotor and Cognitive Impairment: Relevance to Schizophrenia,” Biological Psychiatry 78, no. 11 (2015): 763–774. 25869810 10.1016/j.biopsych.2015.02.026 PMC4714966

[146] I. Paiva , K. Carvalho , P. Santos , et al., “A(2A) R‐Induced Transcriptional Deregulation in Astrocytes: An in Vitro Study,” Glia 67, no. 12 (2019): 2329–2342. 31328322 10.1002/glia.23688

[147] H. S. Lee , A. Ghetti , A. Pinto‐Duarte , et al., “Astrocytes Contribute to Gamma Oscillations and Recognition Memory,” Proceeding of the National Academy of Sciences of the United States of America 111, no. 32 (2014): E3343–3352. 10.1073/pnas.1410893111 PMC4136580 25071179

[148] H. Nishiyama , T. Knopfel , S. Endo , and S. Itohara , “Glial Protein S100B Modulates Long‐Term Neuronal Synaptic Plasticity,” Proceeding of the National Academy of Sciences of the United States of America 99, no. 6 (2002): 4037–4042. 10.1073/pnas.052020999 PMC122644 11891290

[149] V. M. Sardinha , S. Guerra‐Gomes , I. Caetano , et al., “Astrocytic Signaling Supports Hippocampal‐Prefrontal Theta Synchronization and Cognitive Function,” Glia 65, no. 12 (2017): 1944–1960. 28885722 10.1002/glia.23205

[150] A. G. Orr , E. C. Hsiao , M. M. Wang , et al., “Astrocytic Adenosine Receptor A2A and Gs‐Coupled Signaling Regulate Memory,” Nature Neuroscience 18, no. 3 (2015): 423–434. 25622143 10.1038/nn.3930 PMC4340760

[151] K. S. Lee , M. Reddington , P. Schubert , and G. Kreutzberg , “Regulation of the Strength of Adenosine Modulation in the Hippocampus by a Differential Distribution of the Density of A1 Receptors,” Brain Research 260, no. 1 (1983): 156–159. 6297683 10.1016/0006-8993(83)90779-5

[152] S. L. Reis , H. B. Silva , M. Almeida , R. A. Cunha , A. P. Simões , and P. M. Canas , “Adenosine A(1) and A(2A) Receptors Differently Control Synaptic Plasticity in the Mouse Dorsal and Ventral Hippocampus,” Journal of Neurochemistry 151, no. 2 (2019): 227–237. 31274188 10.1111/jnc.14816

[153] Y. Xu , Y. Ning , Y. Zhao , et al., “Caffeine Functions by Inhibiting Dorsal and Ventral Hippocampal Adenosine 2A Receptors to Modulate Memory and Anxiety, Respectively,” Frontiers in Pharmacology 13 (2022): 807330. 35185566 10.3389/fphar.2022.807330 PMC8847668

[154] S. M. Theparambil , O. Kopach , A. Braga , et al., “Adenosine Signalling to Astrocytes Coordinates Brain Metabolism and Function,” Nature 632, no. 8023 (2024): 139–146. 38961289 10.1038/s41586-024-07611-w PMC11291286

[155] C. R. Lopes , R. A. Cunha , and P. Agostinho , “Astrocytes and Adenosine A(2A) Receptors: Active Players in Alzheimer's Disease,” Frontiers in Neuroscience 15 (2021): 666710. 34054416 10.3389/fnins.2021.666710 PMC8155589

[156] A. R. Costenla , M. J. Diógenes , P. M. Canas , et al., “Enhanced Role of Adenosine A(2A) Receptors in the Modulation of LTP in the Rat Hippocampus Upon Ageing,” European Journal of Neuroscience 34, no. 1 (2011): 12–21. 21615561 10.1111/j.1460-9568.2011.07719.x

[157] G. M. A. Cunha , P. M. Canas , C. S. Melo , et al., “Adenosine A2A Receptor Blockade Prevents Memory Dysfunction Caused by Beta‐Amyloid Peptides but Not by Scopolamine or MK‐801,” Experimental Neurology 210, no. 2 (2008): 776–781. 18191838 10.1016/j.expneurol.2007.11.013

[158] P. M. Canas , L. O. Porciúncula , G. M. A. Cunha , et al., “Adenosine A2A Receptor Blockade Prevents Synaptotoxicity and Memory Dysfunction Caused by Beta‐Amyloid Peptides via p38 Mitogen‐Activated Protein Kinase Pathway,” Journal of Neuroscience 29, no. 47 (2009): 14741–14751. 19940169 10.1523/JNEUROSCI.3728-09.2009 PMC6665997

[159] D. Madeira , L. Dias , P. Santos , R. A. Cunha , P. M. Canas , and P. Agostinho , “Association Between Adenosine A(2A) Receptors and Connexin 43 Regulates Hemichannels Activity and ATP Release in Astrocytes Exposed to Amyloid‐β Peptides,” Molecular Neurobiology 58, no. 12 (2021): 6232–6248. 34476674 10.1007/s12035-021-02538-z

[160] L. Dias , D. Pochmann , C. Lemos , et al., “Increased Synaptic ATP Release and CD73‐Mediated Formation of Extracellular Adenosine in the Control of Behavioral and Electrophysiological Modifications Caused by Chronic Stress,” Acs Chemical Neuroscience 14, no. 7 (2023): 1299–1309. 36881648 10.1021/acschemneuro.2c00810 PMC10080657

[161] Y.‐F. Zhao , W.‐J. Ren , Y. Zhang , et al., “High, in Contrast to Low Levels of Acute Stress Induce Depressive‐Like Behavior by Involving Astrocytic, in Addition to Microglial P2×7 Receptors in the Rodent Hippocampus,” International Journal of Molecular Sciences 23, no. 3 (2022): 1904. 35163829 10.3390/ijms23031904 PMC8836505

[162] R. C. Kessler , P. Berglund , O. Demler , et al., “The Epidemiology of Major Depressive Disorder: Results From the National Comorbidity Survey Replication (NCS‐R),” Jama 289, no. 23 (2003): 3095–3105. 12813115 10.1001/jama.289.23.3095

[163] G. S. Malhi and J. J. Mann Lancet 392, no. 10161 (2018): 2299–2312. 30396512 10.1016/S0140-6736(18)31948-2

[164] X.‐J. Kuang , C.‐Y. Zhang , B.‐Y. Yan , et al., “P2×2 Receptors in Pyramidal Neurons Are Critical for Regulating Vulnerability to Chronic Stress,” Theranostics 12, no. 8 (2022): 3703–3718. 35664080 10.7150/thno.72144 PMC9131261

[165] E. Anderzhanova , T. Kirmeier , and C. T. Wotjak , “Animal Models in Psychiatric Research: The RDoC System as a New Framework for Endophenotype‐Oriented Translational Neuroscience,” Neurobiology of Stress 7 (2017): 47–56. 28377991 10.1016/j.ynstr.2017.03.003 PMC5377486

[166] C. R. Pryce and E. Fuchs , “Chronic Psychosocial Stressors in Adulthood: Studies in Mice, Rats and Tree Shrews,” Neurobiology of Stress 6 (2017): 94–103. 28229112 10.1016/j.ynstr.2016.10.001 PMC5314423

[167] J. W. Young , B. L. Henry , and M. A. Geyer , “Predictive Animal Models of Mania: Hits, Misses and Future Directions,” British Journal of Pharmacology 164, no. 4 (2011): 1263–1284. 21410454 10.1111/j.1476-5381.2011.01318.x PMC3229761

[168] D. Rial , C. Lemos , H. Pinheiro , et al., “Depression as a Glial‐Based Synaptic Dysfunction,” Frontiers in Cellular Neuroscience 9 (2015): 521. 26834566 10.3389/fncel.2015.00521 PMC4722129

[169] K. Harada , T. Kamiya , and T. Tsuboi , “Gliotransmitter Release From Astrocytes: Functional, Developmental, and Pathological Implications in the Brain,” Frontiers in Neuroscience 9 (2015): 499. 26793048 10.3389/fnins.2015.00499 PMC4709856

[170] S. Koizumi , “Glial Purinergic Signals and Psychiatric Disorders,” Frontiers in Cellular Neuroscience 15 (2021): 822614. 35069121 10.3389/fncel.2021.822614 PMC8766327

[171] Q.‐Q. Cui , Z.‐L. Hu , Y.‐L. Hu , et al., “Hippocampal CD39/ENTPD1 Promotes Mouse Depression‐Like Behavior Through Hydrolyzing Extracellular ATP,” Embo Reports 21, no. 4 (2020): e47857. 32133764 10.15252/embr.201947857 PMC7132197

[172] S. Lin , L. Huang , Z.‐C. Luo , et al., “The ATP Level in the Medial Prefrontal Cortex Regulates Depressive‐Like Behavior via the Medial Prefrontal Cortex‐Lateral Habenula Pathway,” Biological Psychiatry 92, no. 3 (2022): 179–192. 35489874 10.1016/j.biopsych.2022.02.014

[173] M. Kinoshita , Y. Hirayama , K. Fujishita , et al., “Anti‐Depressant Fluoxetine Reveals Its Therapeutic Effect via Astrocytes,” EBioMedicine 32 (2018): 72–83. 29887330 10.1016/j.ebiom.2018.05.036 PMC6020856

[174] A. R. Machado‐Santos , E. Loureiro‐Campos , P. Patrício , et al., “Beyond New Neurons in the Adult Hippocampus: Imipramine Acts as a Pro‐Astrogliogenic Factor and Rescues Cognitive Impairments Induced by Stress Exposure,” Cells 11, no. 3 (2022): 390. 35159199 10.3390/cells11030390 PMC8834148

[175] H. N. Rubaiy , “ORAI Calcium Channels: Regulation, Function, Pharmacology, and Therapeutic Targets,” Pharmaceuticals (Basel) 16, no. 2 (2023): 162. 37259313 10.3390/ph16020162 PMC9967976

[176] M. M. Novakovic , K. S. Korshunov , R. A. Grant , et al., “Astrocyte Reactivity and Inflammation‐Induced Depression‐Like Behaviors Are Regulated by Orai1 Calcium Channels,” Nature Communications 14, no. 1 (2023): 5500. 10.1038/s41467-023-40968-6 PMC10485021 37679321

[177] J. Liu , T.‐T. Liu , L. Mou , et al., “P2×7 Receptor: A Potential Target for Treating Comorbid Anxiety and Depression,” Purinergic Signalling (2024). 10.1007/s11302-024-10007-0 PMC12454246 38642324

[178] W.‐H. Cho , K. Noh , B. H. Lee , et al., “Hippocampal Astrocytes Modulate Anxiety‐Like Behavior,” Nature Communications 13, no. 1 (2022): 6536. 10.1038/s41467-022-34201-z PMC9640657 36344520

[179] Q. Ren , Z.‐Z. Wang , S.‐F. Chu , C.‐Y. Xia , and N.‐H. Chen , “Gap Junction Channels as Potential Targets for the Treatment of Major Depressive Disorder,” Psychopharmacology 235, no. 1 (2018): 1–12. 29178009 10.1007/s00213-017-4782-7

[180] C.‐Y. Xia , Z.‐Z. Wang , T. Yamakuni , and N.‐H. Chen , “A Novel Mechanism of Depression: Role for Connexins,” European Neuropsychopharmacology 28, no. 4 (2018): 483–498. 29519610 10.1016/j.euroneuro.2018.01.009

[181] D. Huang , C. Li , W. Zhang , J. Qin , W. Jiang , and C. Hu , “Dysfunction of Astrocytic Connexins 30 and 43 in the Medial Prefrontal Cortex and Hippocampus Mediates Depressive‐Like Behaviours,” Behavioural Brain Research 372 (2019): 111950. 31103752 10.1016/j.bbr.2019.111950

[182] M. Ni , J.‐G. He , H.‐Y. Zhou , et al., “Pannexin‐1 Channel Dysfunction in the Medial Prefrontal Cortex Mediates Depressive‐Like Behaviors Induced by Chronic Social Defeat Stress and Administration of Mefloquine in Mice,” Neuropharmacology 137 (2018): 256–267. 29221793 10.1016/j.neuropharm.2017.12.004

[183] J. A. Orellana , R. Moraga‐Amaro , R. Díaz‐Galarce , et al., “Restraint Stress Increases Hemichannel Activity in Hippocampal Glial Cells and Neurons,” Frontiers in Cellular Neuroscience 9 (2015): 102. 25883550 10.3389/fncel.2015.00102 PMC4382970

[184] M. Jun , Q. Xiaolong , Y. Chaojuan , et al., “Calhm2 Governs Astrocytic ATP Releasing in the Development of Depression‐Like Behaviors,” Molecular Psychiatry 23, no. 4 (2018): 1091. 29311664 10.1038/mp.2017.254

[185] T. Hajszan , A. Dow , J. L. Warner‐Schmidt , et al., “Remodeling of Hippocampal Spine Synapses in the Rat Learned Helplessness Model of Depression,” Biological Psychiatry 65, no. 5 (2009): 392–400. 19006787 10.1016/j.biopsych.2008.09.031 PMC2663388

[186] Y. Liao , Y. Wang , Q.‐Q. Tao , et al., “CALHM2 V136G Polymorphism Reduces Astrocytic ATP Release and Is Associated With Depressive Symptoms and Alzheimer's Disease Risk,” Alzheimer's & Dementia 19, no. 10 (2023): 4407–4420. 10.1002/alz.13366 37493186

[187] M. Iwata , K. T. Ota , X.‐Y. Li , et al., “Psychological Stress Activates the Inflammasome via Release of Adenosine Triphosphate and Stimulation of the Purinergic Type 2×7 Receptor,” Biological Psychiatry 80, no. 1 (2016): 12–22. 26831917 10.1016/j.biopsych.2015.11.026

[188] N. Yue , H. Huang , X. Zhu , et al., “Activation of P2×7 Receptor and NLRP3 Inflammasome Assembly in Hippocampal Glial Cells Mediates Chronic Stress‐Induced Depressive‐Like Behaviors,” Journal of Neuroinflammation 14, no. 1 (2017): 102. 28486969 10.1186/s12974-017-0865-y PMC5424302

[189] M. Ma , Q. Ren , J.‐C. Zhang , and K. Hashimoto , “Effects of Brilliant Blue G on Serum Tumor Necrosis Factor‐α Levels and Depression‐Like Behavior in Mice After Lipopolysaccharide Administration,” Clinical Psychopharmacology and Neuroscience 12, no. 1 (2014): 31–36. 24851118 10.9758/cpn.2014.12.1.31 PMC4022763

[190] E. Akcay and H. Karatas , “P2×7 Receptors From the Perspective of NLRP3 Inflammasome Pathway in Depression: Potential Role of Cannabidiol,” Brain, Behavior, & Immunity ‐ Health 41 (2024): 100853. 10.1016/j.bbih.2024.100853 PMC11407962 39296605

[191] G. Ghaffaripour Jahromi , S. Razi , and N. Rezaei , “NLRP3 Inflammatory Pathway. Can We Unlock Depression?,” Brain Research 1822 (2024): 148644. 37871673 10.1016/j.brainres.2023.148644

[192] M. Xia , Z. Li , S. Li , et al., “Sleep Deprivation Selectively Down‐Regulates Astrocytic 5‐HT(2B) Receptors and Triggers Depressive‐Like Behaviors via Stimulating P2X(7) Receptors in Mice,” Neuroscience Bulletin 36, no. 11 (2020): 1259–1270. 32506374 10.1007/s12264-020-00524-4 PMC7674526

[193] Y. Nishioka , K. Hayashi , K. Morito , K. Takayama , and K. Nagasawa , “Altered Expression of Astrocytic ATP Channels and Ectonucleotidases in the Cerebral Cortex and Hippocampus of Chronic Social Defeat Stress‐Susceptible BALB/c Mice,” Biological & Pharmaceutical Bulletin 47, no. 6 (2024): 1172–1178. 38880625 10.1248/bpb.b24-00236

[194] W. Xiong , X. Cao , Y. Zeng , et al., “Astrocytic Epoxyeicosatrienoic Acid Signaling in the Medial Prefrontal Cortex Modulates Depressive‐Like Behaviors,” Journal of Neuroscience 39, no. 23 (2019): 4606–4623. 30902874 10.1523/JNEUROSCI.3069-18.2019 PMC6554635

[195] Q. Ren , M. Ma , T. Ishima , et al., “Gene Deficiency and Pharmacological Inhibition of Soluble Epoxide Hydrolase Confers Resilience to Repeated Social Defeat Stress,” Proceeding of the National Academy of Sciences of the United States of America 113, no. 13 (2016): E1944–1952. 10.1073/pnas.1601532113 PMC4822604 26976569

[196] K. W. Lee , S. M. Ching , V. Ramachandran , et al., “Association Analysis of 14 Candidate Gene Polymorphism With Depression and Stress Among Gestational Diabetes Mellitus,” Genes (Basel) 10, no. 12 (2019): 988. 31801286 10.3390/genes10120988 PMC6947641

[197] Y. Moriyama , M. Hiasa , S. Sakamoto , H. Omote , and M. Nomura , “Vesicular Nucleotide Transporter (VNUT): Appearance of an Actress on the Stage of Purinergic Signaling,” Purinergic Signalling 13, no. 3 (2017): 387–404. 28616712 10.1007/s11302-017-9568-1 PMC5563297

[198] Y. Kato , K. Ohsugi , Y. Fukuno , K. Iwatsuki , Y. Harada , and T. Miyaji , “Vesicular Nucleotide Transporter Is a Molecular Target of Eicosapentaenoic Acid for Neuropathic and Inflammatory Pain Treatment,” Proceeding of the National Academy of Sciences of the United States of America 119, no. 30 (2022): e2122158119. 10.1073/pnas.2122158119 PMC9335333 35858418

[199] Y. Liao , B. Xie , H. Zhang , et al., “Efficacy of Omega‐3 PUFAs in Depression: A Meta‐Analysis,” Translational Psychiatry 9, no. 1 (2019): 190. 31383846 10.1038/s41398-019-0515-5 PMC6683166

[200] C. Gubert , G. R. Fries , B. Pfaffenseller , et al., “Role of P2×7 Receptor in an Animal Model of Mania Induced by D‐Amphetamine,” Molecular Neurobiology 53, no. 1 (2016): 611–620. 25502294 10.1007/s12035-014-9031-z

[201] C. Gubert , R. Andrejew , C. E. Leite , et al., “P2×7 Purinergic Receptor Is Involved in the Pathophysiology of Mania: A Preclinical Study,” Molecular Neurobiology 57, no. 3 (2020): 1347–1360. 31729632 10.1007/s12035-019-01817-0

[202] C. Csölle , R. D. Andó , Á. Kittel , et al., “The Absence of P2×7 Receptors (P2rx7) on Non‐Haematopoietic Cells Leads to Selective Alteration in Mood‐Related Behaviour With Dysregulated Gene Expression and Stress Reactivity in Mice,” The International Journal of Neuropsychopharmacology 16, no. 1 (2013): 213–233. 22243662 10.1017/S1461145711001933 PMC3666310

[203] F. Gölöncsér , M. Baranyi , P. Tod , F. Maácz , and B. Sperlágh , “P2×7 Receptor Inhibition Alleviates Mania‐Like Behavior Independently of Interleukin‐1β,” Iscience 27, no. 3 (2024): 109284. 38444608 10.1016/j.isci.2024.109284 PMC10914489

[204] P. Agostinho , D. Madeira , L. Dias , A. P. Simões , R. A. Cunha , and P. M. Canas , “Purinergic Signaling Orchestrating Neuron‐Glia Communication,” Pharmacological Research 162 (2020): 105253. 33080321 10.1016/j.phrs.2020.105253

[205] D. van Calker , K. Biber , K. Domschke , and T. Serchov , “The Role of Adenosine Receptors in Mood and Anxiety Disorders,” Journal of Neurochemistry 151, no. 1 (2019): 11–27. 31361031 10.1111/jnc.14841

[206] J. I. Gomes , M. Farinha‐Ferreira , N. Rei , et al., “Of Adenosine and the Blues: The Adenosinergic System in the Pathophysiology and Treatment of Major Depressive Disorder,” Pharmacological Research 163 (2021): 105363. 33285234 10.1016/j.phrs.2020.105363

[207] S. Pasquini , C. Contri , S. Merighi , et al., “Adenosine Receptors in Neuropsychiatric Disorders: Fine Regulators of Neurotransmission and Potential Therapeutic Targets,” International Journal of Molecular Sciences 23, no. 3 (2022): 1219. 35163142 10.3390/ijms23031219 PMC8835915

[208] M. P. Kaster , N. J. Machado , H. B. Silva , et al., “Caffeine Acts Through Neuronal Adenosine A2A Receptors to Prevent Mood and Memory Dysfunction Triggered by Chronic Stress,” Proceeding of the National Academy of Sciences of the United States of America 112, no. 25 (2015): 7833–7838. 10.1073/pnas.1423088112 PMC4485143 26056314

[209] T. Serchov , I. Schwarz , A. Theiss , et al., “Enhanced Adenosine A(1) Receptor and Homer1a Expression in Hippocampus Modulates the Resilience to Stress‐Induced Depression‐Like Behavior,” Neuropharmacology 162 (2020): 107834. 31682853 10.1016/j.neuropharm.2019.107834

[210] A. Camargo , L. E. B. Bettio , P. B. Rosa , J. M. Rosa , G. A. Altê , and A. L. S. Rodrigues , “The Antidepressant‐Like Effect of Guanosine Involves the Modulation of Adenosine A(1) and A(2A) Receptors,” Purinergic Signalling 19, no. 2 (2023): 387–399. 36166131 10.1007/s11302-022-09898-8 PMC10247657

[211] T. Kroll , M. Grözinger , A. Matusch , et al., “Effects of Electroconvulsive Therapy on Cerebral A(1) Adenosine Receptor Availability: A PET Study in Patients Suffering From Treatment‐Resistant Major Depressive Disorder,” Frontiers in Psychiatry 14 (2023): 1228438. 37520217 10.3389/fpsyt.2023.1228438 PMC10380952

[212] V. Lazarevic , Y. Yang , I. Flais , and P. Svenningsson , “Ketamine Decreases Neuronally Released Glutamate via Retrograde Stimulation of Presynaptic Adenosine A1 Receptors,” Molecular Psychiatry 26, no. 12 (2021): 7425–7435. 34376822 10.1038/s41380-021-01246-3 PMC8872981

[213] Q. Guo , D. Gobbo , N. Zhao , et al., “Adenosine Triggers Early Astrocyte Reactivity That Provokes Microglial Responses and Drives the Pathogenesis of Sepsis‐Associated Encephalopathy in Mice,” Nature Communications 15, no. 1 (2024): 6340. 10.1038/s41467-024-50466-y PMC11283516 39068155

[214] S. Sharma , S. Chawla , P. Kumar , R. Ahmad , and P. Kumar Verma , “The Chronic Unpredictable Mild Stress (CUMS) Paradigm: Bridging the Gap in Depression Research From Bench to Bedside,” Brain Research 1843 (2024): 149123. 39025397 10.1016/j.brainres.2024.149123

[215] M. Wang , P. Li , Z. Li , et al., “Lateral Septum Adenosine A(2A) Receptors Control Stress‐Induced Depressive‐Like Behaviors via Signaling to the Hypothalamus and Habenula,” Nature Communications 14, no. 1 (2023): 1880. 10.1038/s41467-023-37601-x PMC10076302 37019936

[216] X.‐J. Lv , S.‐S. Lv , G.‐H. Wang , et al., “Glia‐Derived Adenosine in the Ventral Hippocampus Drives Pain‐Related Anxiodepression in a Mouse Model Resembling Trigeminal Neuralgia,” Brain, Behavior, and Immunity 117 (2024): 224–241. 38244946 10.1016/j.bbi.2024.01.012

[217] G. Burnstock , U. Krügel , M. P. Abbracchio , and P. Illes , “Purinergic Signalling: From Normal Behaviour to Pathological Brain Function,” Progress in Neurobiology 95, no. 2 (2011): 229–274. 21907261 10.1016/j.pneurobio.2011.08.006

[218] R. Bartlett , L. Stokes , and R. Sluyter , “The P2×7 Receptor Channel: Recent Developments and the Use of P2×7 Antagonists in Models of Disease,” Pharmacological Reviews 66, no. 3 (2014): 638–675. 24928329 10.1124/pr.113.008003

[219] B. Sperlágh and P. Illes , “P2×7 Receptor: An Emerging Target in Central Nervous System Diseases,” Trends in Pharmacological Sciences 35, no. 10 (2014): 537–547. 25223574 10.1016/j.tips.2014.08.002

[220] D. E. Ribeiro , L. L. Petiz , T. Glaser , et al., “Purinergic Signaling in Cognitive Impairment and Neuropsychiatric Symptoms of Alzheimer's Disease,” Neuropharmacology 226 (2023): 109371. 36502867 10.1016/j.neuropharm.2022.109371

[221] C. Pizzirani , D. Ferrari , P. Chiozzi , et al., “Stimulation of P2 Receptors Causes Release of IL‐1beta‐Loaded Microvesicles From Human Dendritic Cells,” Blood 109, no. 9 (2007): 3856–3864. 17192399 10.1182/blood-2005-06-031377

[222] G. Conte , A. Menéndez‐Méndez , S. Bauer , et al., “Circulating P2×7 Receptor Signaling Components as Diagnostic Biomarkers for Temporal Lobe Epilepsy,” Cells 10, no. 9 (2021): 2444. 34572093 10.3390/cells10092444 PMC8467140

[223] J. García‐Villalba , L. Hurtado‐Navarro , A. Peñín‐Franch , et al., “Soluble P2×7 Receptor Is Elevated in the Plasma of COVID‐19 Patients and Correlates With Disease Severity,” Frontiers in Immunology 13 (2022): 894470. 35663992 10.3389/fimmu.2022.894470 PMC9161710

[224] F. Di Virgilio , V. Vultaggio‐Poma , S. Falzoni , and A. L. Giuliani , “The Coming of Age of the P2×7 Receptor in Diagnostic Medicine,” International Journal of Molecular Sciences 24, no. 11 (2023): 9465. 37298415 10.3390/ijms24119465 PMC10253666

[225] P. Aivar , C. Bianchi , C. Di Lauro , et al., “TNAP and P2×7R: New Plasma Biomarkers for Alzheimer's Disease,” International Journal of Molecular Sciences 24, no. 13 (2023): 10897. 37446074 10.3390/ijms241310897 PMC10342008

[226] A. L. Giuliani , M. Berchan , J. M. Sanz , et al., “The P2×7 Receptor Is Shed Into Circulation: Correlation With C‐Reactive Protein Levels,” Frontiers in Immunology 10 (2019): 793. 31031771 10.3389/fimmu.2019.00793 PMC6474289

[227] C. N. J. Young and D. C. Górecki , “P2RX7 Purinoceptor as a Therapeutic Target‐The Second Coming?,” Frontiers in Chemistry 6 (2018): 248. 30003075 10.3389/fchem.2018.00248 PMC6032550

[228] Q. H. Zheng , “Radioligands Targeting Purinergic P2×7 Receptor,” Bioorganic & Medicinal Chemistry Letters 30, no. 12 (2020): 127169. 32273217 10.1016/j.bmcl.2020.127169

[229] S. Schmidt , A. Isaak , and A. Junker , “Spotlight on P2×7 Receptor PET Imaging: A Bright Target or a Failing Star?,” International Journal of Molecular Sciences 24, no. 2 (2023): 1374. 36674884 10.3390/ijms24021374 PMC9861945

[230] R. H. Mach , “PET Imaging of Microglial Activation‐Beyond Targeting TSPO,” Molecules (Basel, Switzerland) 23, no. 3 (2018): 607. 29518005 10.3390/molecules23030607 PMC6017265

[231] J. H. Meyer , S. Cervenka , M.‐J. Kim , W. C. Kreisl , I. D. Henter , and R. B. Innis , “Neuroinflammation in Psychiatric Disorders: PET Imaging and Promising New Targets,” Lancet Psychiatry 7, no. 12 (2020): 1064–1074. 33098761 10.1016/S2215-0366(20)30255-8 PMC7893630

[232] M. H. J. Hagens , S. S. V. Golla , B. Janssen , et al., “The P2X(7) Receptor Tracer (11)CSMW139 as an in Vivo Marker of Neuroinflammation in Multiple Sclerosis: A First‐In Man Study,” European Journal of Nuclear Medicine and Molecular Imaging 47, no. 2 (2020): 379–389. 31705174 10.1007/s00259-019-04550-x PMC6974509

[233] M. Wang , “Characterization of (11)C‐GSK1482160 for Targeting the P2×7 Receptor as a Biomarker for Neuroinflammation,” Journal of Nuclear Medicine 58, no. 3 (2017): 458–465. 27765863 10.2967/jnumed.116.181354

[234] J. D. Mikkelsen , S. S. Aripaka , S. Kaad , et al., “Characterization of the Novel P2×7 Receptor Radioligand (3)HJNJ‐64413739 in Human Brain Tissue,” Acs Chemical Neuroscience 14, no. 1 (2023): 111–118. 36535632 10.1021/acschemneuro.2c00561 PMC9817075

[235] E. de Marchi , E. Orioli , D. Dal Ben , and E. Adinolfi , “P2×7 Receptor as a Therapeutic Target,” Advances in Protein Chemistry and Structural Biology 104 (2016): 39–79. 27038372 10.1016/bs.apcsb.2015.11.004

[236] X. Liu , Y. Li , L. Huang , et al., “Unlocking the Therapeutic Potential of P2×7 Receptor: A Comprehensive Review of Its Role in Neurodegenerative Disorders,” Frontiers in Pharmacology 15 (2024): 1450704. 39139642 10.3389/fphar.2024.1450704 PMC11319138

[237] E. C. Keystone , M. M. Wang , M. Layton , S. Hollis , and I. B. McInnes , “Clinical Evaluation of the Efficacy of the P2×7 Purinergic Receptor Antagonist AZD9056 on the Signs and Symptoms of Rheumatoid Arthritis in Patients With Active Disease Despite Treatment With Methotrexate or Sulphasalazine,” Annals of the Rheumatic Diseases 71, no. 10 (2012): 1630–1635. 22966146 10.1136/annrheumdis-2011-143578

[238] T. C. Stock , B. J. Bloom , N. Wei , et al., “Efficacy and Safety of CE‐224,535, an Antagonist of P2×7 Receptor, in Treatment of Patients With Rheumatoid Arthritis Inadequately Controlled by Methotrexate,” Journal of Rheumatology 39, no. 4 (2012): 720–727. 22382341 10.3899/jrheum.110874

[239] A. Eser , J.‐F. Colombel , P. Rutgeerts , et al., “Safety and Efficacy of an Oral Inhibitor of the Purinergic Receptor P2×7 in Adult Patients With Moderately to Severely Active Crohn's Disease: A Randomized Placebo‐Controlled, Double‐Blind, Phase IIa Study,” Inflammatory Bowel Diseases 21, no. 10 (2015): 2247–2253. 26197451 10.1097/MIB.0000000000000514

[240] J. C. Rech , A. Bhattacharya , M. A. Letavic , and B. M. Savall , “The Evolution of P2×7 Antagonists With a Focus on CNS Indications,” Bioorganic & Medicinal Chemistry Letters 26, no. 16 (2016): 3838–3845. 27426304 10.1016/j.bmcl.2016.06.048

[241] K. Recourt , P. de Boer , P. van der Ark , et al., “Characterization of the Central Nervous System Penetrant and Selective Purine P2×7 Receptor Antagonist JNJ‐54175446 in Patients With Major Depressive Disorder,” Translational Psychiatry 13, no. 1 (2023): 266. 37482560 10.1038/s41398-023-02557-5 PMC10363543

[242] A. Bhattacharya and D. N. C. Jones , “Emerging Role of the P2×7‐NLRP3‐IL1β Pathway in Mood Disorders,” Psychoneuroendocrinology 98 (2018): 95–100. 30121550 10.1016/j.psyneuen.2018.08.015

[243] A. Bhattacharya , Q. Wang , H. Ao , et al., “Pharmacological Characterization of a Novel Centrally Permeable P2×7 Receptor Antagonist: JNJ‐47965567,” British Journal of Pharmacology 170, no. 3 (2013): 624–640. 23889535 10.1111/bph.12314 PMC3792000

[244] T. Stähler , W. Danquah , M. Demeules , et al., “Development of Antibody and Nanobody Tools for P2×7,” Methods in Molecular Biology 2510 (2022): 99–127. 35776322 10.1007/978-1-0716-2384-8_6

[245] F. Koch‐Nolte , “Nanobody‐Based Heavy Chain Antibodies and Chimeric Antibodies,” Immunological Reviews 328, no. 1 (2024): 466–472. 39212236 10.1111/imr.13385 PMC11659929

[246] H. Lee , A. Elkamhawy , P. Rakhalskaya , et al., “Small Molecules in Parkinson's Disease Therapy: From Dopamine Pathways to New Emerging Targets,” Pharmaceuticals (Basel) 17, no. 12 (2024): 1688. 39770531 10.3390/ph17121688 PMC11677913

[247] L. Sequeira , S. Benfeito , C. Fernandes , et al., “Drug Development for Alzheimer's and Parkinson's Disease: Where Do We Go Now?,” Pharmaceutics 16, no. 6 (2024): 708. 38931832 10.3390/pharmaceutics16060708 PMC11206728

[248] X. Qi , D. Nizamutdinov , S. S. Yi , E. Wu , and J. H. Huang , “Disease Modifying Monoclonal Antibodies and Symptomatic Pharmacological Treatment for Alzheimer's Disease,” Biomedicines 12, no. 11 (2024): 2636. 39595200 10.3390/biomedicines12112636 PMC11592475

[249] F. Mirzaei , L. Agbaria , K. Bhatnagar , et al., “Coffee and Alzheimer's Disease,” Progress in Brain Research 289 (2024): 21–55. 39168581 10.1016/bs.pbr.2024.06.002

[250] S. Merighi , P. A. Borea , K. Varani , F. Vincenzi , K. A. Jacobson , and S. Gessi , “A(2A) Adenosine Receptor Antagonists in Neurodegenerative Diseases,” Current Medicinal Chemistry 29, no. 24 (2022): 4138–4151. 34844537 10.2174/0929867328666211129122550 PMC9148371

[251] S. H. Isaacson , S. Betté , and R. Pahwa , “Istradefylline for OFF Episodes in Parkinson's Disease: A US Perspective of Common Clinical Scenarios,” Degenerative Neurological and Neuromuscular Disease 12 (2022): 97–109. 35910426 10.2147/DNND.S245197 PMC9329678

[252] J.‐F. Chen and R. A. Cunha , “The Belated US FDA Approval of the Adenosine A(2A) Receptor Antagonist Istradefylline for Treatment of Parkinson's Disease,” Purinergic Signalling 16, no. 2 (2020): 167–174. 32236790 10.1007/s11302-020-09694-2 PMC7367999

[253] S. Ferré , J. Bonaventura , D. Tomasi , et al., “Allosteric Mechanisms Within the Adenosine A2A‐Dopamine D2 Receptor Heterotetramer,” Neuropharmacology 104 (2016): 154–160. 26051403 10.1016/j.neuropharm.2015.05.028 PMC5754196

[254] K. R. Chaudhuri , D. G. Healy , and A. H. V. Schapira , “Non‐Motor Symptoms of Parkinson's Disease: Diagnosis and Management,” Lancet Neurology 5, no. 3 (2006): 235–245. 16488379 10.1016/S1474-4422(06)70373-8

[255] S. Uchida , T. Kadowaki‐Horita , and T. Kanda , “Effects of the Adenosine A2A Receptor Antagonist on Cognitive Dysfunction in Parkinson's Disease,” International Review of Neurobiology 119 (2014): 169–189. 25175966 10.1016/B978-0-12-801022-8.00008-8

